# Dairy byproducts as sustainable alternatives to FCS in 2D and 3D skeletal muscle cell cultures

**DOI:** 10.1186/s40643-025-00938-w

**Published:** 2025-09-22

**Authors:** Tobias Horst Baldeweg, Philipp Hubel, Johannes Günther, Fabian Ostertag, Svenja Nellinger, Simon Heine, Petra Juliane Kluger

**Affiliations:** 1 Reutlingen Research Institute, Reutlingen University, 72762 Reutlingen, Germany; 2 Core Facility Hohenheim, Mass Spectrometry Core Facility, University of Hohenheim, 70599 Stuttgart, Germany; 3 Core Facility Hohenheim, Spectroscopy Unit, University of Hohenheim, 70599 Stuttgart, Germany; 4 Institute of Food Science and Biotechnology, Department of Soft Matter Science and Dairy Technology, University of Hohenheim, 70599 Stuttgart, Germany; 5 Institute of Interfacial Process Engineering and Plasma Technology (IGVP), 70569 Stuttgart, Germany; 6 Fraunhofer Institute for Interfacial Engineering and Biotechnology IGB, 70569 Stuttgart, Germany; 7 School of Life Sciences, Reutlingen University, 72762 Reutlingen, Germany

**Keywords:** Serum-free, Colostrum, Whey, C2C12, Myosphere

## Abstract

Skeletal muscle tissue engineering is a rapidly developing field with applications in disease modelling, tissue replacement, biorobotics, and cultivated meat. The need for more sustainable and ethical biotechnologies has grown due to concerns about resource scarcity, climate change, and animal welfare. One major challenge is replacing fetal calf serum (FCS), a widely used but ethically and environmentally highly problematic media supplement. A promising alternative is the utilization of natural byproducts such as whey and colostrum from the dairy industry, which provide essential nutrients and growth factors. In this study, wheys produced by microfiltration of raw milk and colostrum were investigated as FCS replacements for culturing C2C12 skeletal muscle cells. Composition analysis confirmed a variety of pro-proliferative compounds in both substances. Cell culture experiments led to the development of an optimized medium formulation based on colostrum whey. Colostrum whey medium (CM) supported cell proliferation and maintained the myogenic differentiation potential for over 30 days. Additionally, a CM-based freezing solution enabled effective cryopreservation throughout culture. In 3D static suspension culture, CM sustained viable spheroids for over 14 days. Spheroids showed significantly higher proliferation compared to those in serum-containing medium, making CM suitable for 3D modelling and scale-up of biomass production. These findings highlight CM as a sustainable, cost-effective, and ethical alternative for skeletal muscle tissue engineering, particularly in cultivated meat production.

**Graphical Abstract:**

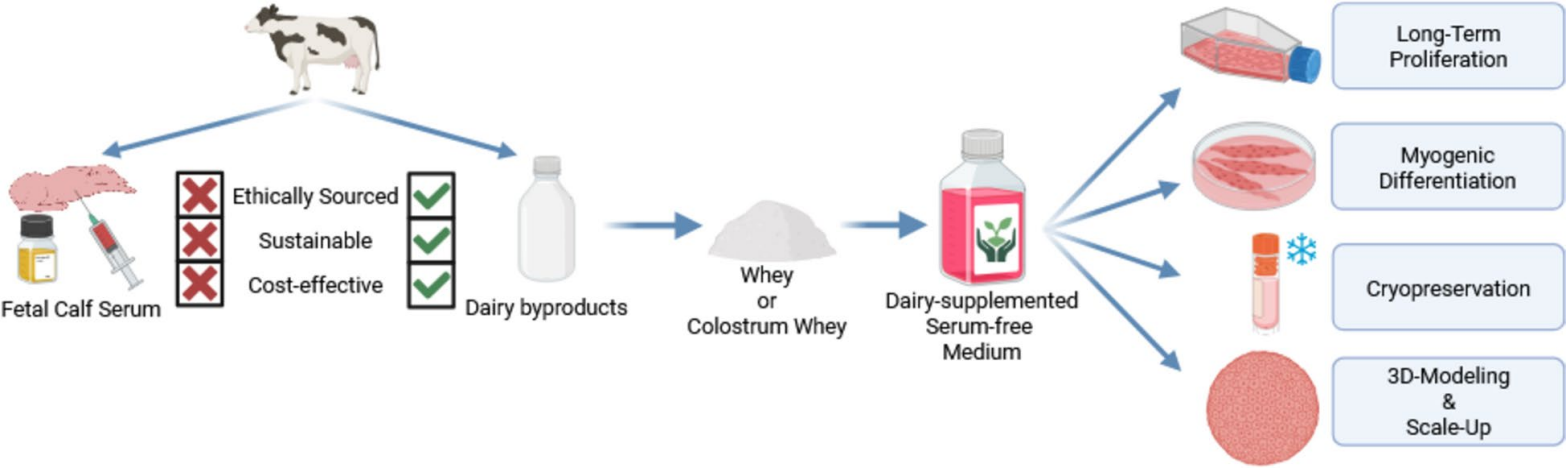

**Supplementary Information:**

The online version contains supplementary material available at 10.1186/s40643-025-00938-w.

## Introduction

As a multidisciplinary field bridging medicine, engineering, and life sciences, tissue engineering combines this expertise to recreate functional tissue to substitute damaged or malfunctioning ones (Langer and Vacanti 1993). Among other organs, skeletal muscle has become a major target in tissue engineering due to its significance in medicine, society, and the environment. Tissue-engineered muscle aids in understanding and treating diseases such as Duchenne muscular dystrophy, and repairing volumetric muscle loss (Shoji et al. 2015; Nesmith et al. 2016; Choi et al. 2019; Niknezhad et al. 2024). Furthermore, the same techniques extent to more unconventional ideas, such as bio-robotics or cultivated meat. Bio-robots utilize muscle tissue instead of traditional actuators, such as pneumatic systems. This allows for smaller robots capable of high force generation and self-healing (Raman et al. 2017; Ricotti et al. 2017; Filippi et al. 2022). Cultivated meat focuses on growing muscle tissue in bioreactors for human consumption. Its goal is to mimic texture and nutritional value of conventional meat rather than tissue functionality (Broucke et al. 2023). This approach addresses food security sustainably and reduces environmental impact. Cultivated meat offers a lower carbon footprint, conserves land and water, and promotes animal welfare by eliminating slaughter (Sinke et al. 2023).

Great efforts have been made to increase the sustainability of tissue engineering, including the use of plant-based materials, food waste, and even human waste as biomaterials (Allan et al. 2021; Zhang et al. 2022a; Farshidfar et al. 2023; Nath et al. 2024; Filippi et al. 2025). Nonetheless, fetal calf serum (FCS) remains widely used in research. FCS contains a variety of nutrients and bioactive proteins, including growth factors, hormones, and cell attachment factors, which have made it the gold standard in cell culture for a long time (Subbiahanadar Chelladurai et al. 2021). However, its production raises sustainability, ethical, and animal welfare concerns. The serum is obtained by blood extraction via cardiac puncture of bovine fetuses after the slaughter of the mother cow. The process involves intensive husbandry and slaughter practices. Furthermore, it could be linked to immense pain for the fetus since the fetal capacity to experience pain during blood collection remains uncertain (McCann and Treasure 2022).

This clearly demonstrates the demand for sustainable, serum-free culture methods in tissue engineering, particularly in cultivated meat. Various approaches have been tested for skeletal muscle cells and progenitors, including chemically defined media and supplementation with extracts from sustainable resources such as microalgae, rapeseed, earthworms, or cyanobacteria (Kolkmann et al. 2022; Stout et al. 2022, 2023; Defendi-Cho and Gould 2023; Ghosh et al. 2023; Morikura et al. 2024; Rossan Mathews et al. 2024). Following the idea of recycling, byproducts and waste from the food industry, such as eggshell membrane, yeast extracts, and pork plasma have also been evaluated as supplements for serum-free myoblast culture (Andreassen et al. 2020).

The dairy industry offers a potential source for recycling high amounts of insufficiently valorized byproducts, such as whey, colostrum, cream, or buttermilk (Banaszewska et al. 2014). These byproducts are often discarded, negatively affecting the environment (Pereira et al. 2015; Simon et al. 2022). Whey from cheese making, colostrum, and milk serum show particularly promise as serum alternatives in cell culture. With about 85–90% of the total milk volume, whey contains a complex mix of lactose, bioactive proteins, vitamins, minerals, and trace elements (Skryplonek et al. 2019; Olvera-Rosales et al. 2023). Its main proteins include β-lactoglobulin (β-LG), α-lactalbumin (α-LA), immunoglobulins, serum albumins (SA), and lactoferrin, along with growth factors such as insulin-like growth factor (IGF), transforming growth factor β (TGF-β), epidermal growth factor (EGF), and fibroblast growth factor (FGF) (Patel 2015; Pouliot and Gauthier 2006). Recent studies demonstrated that whey from ultrafiltration, as well as mixes of three whey proteins (β-LG, α-LA, SA), support short-term serum-free culture of C2C12 cells and bovine satellite cells for up to 10 days (Shima et al. 2025; Sundaram et al. 2025). However, no dairy-based medium has been described for long-term or 3D skeletal myoblast culture.

This study investigates whey fractions derived from milk and colostrum via microfiltration, analyzing their composition and potential applications in cell culture. Tested on C2C12 myoblasts, these fractions enabled the development of a serum-free medium, which supported proliferation and the myogenic differentiation potential in long-term culture over 30 days. Additionally, the compatibility with cryopreservation and 3D spheroid suspension culture over 14 days was confirmed, highlighting its potential for skeletal muscle tissue engineering and cultivated meat production.

## Material and methods

### Separation of the serum phase from milk and colostrum

**Fig. 1:**
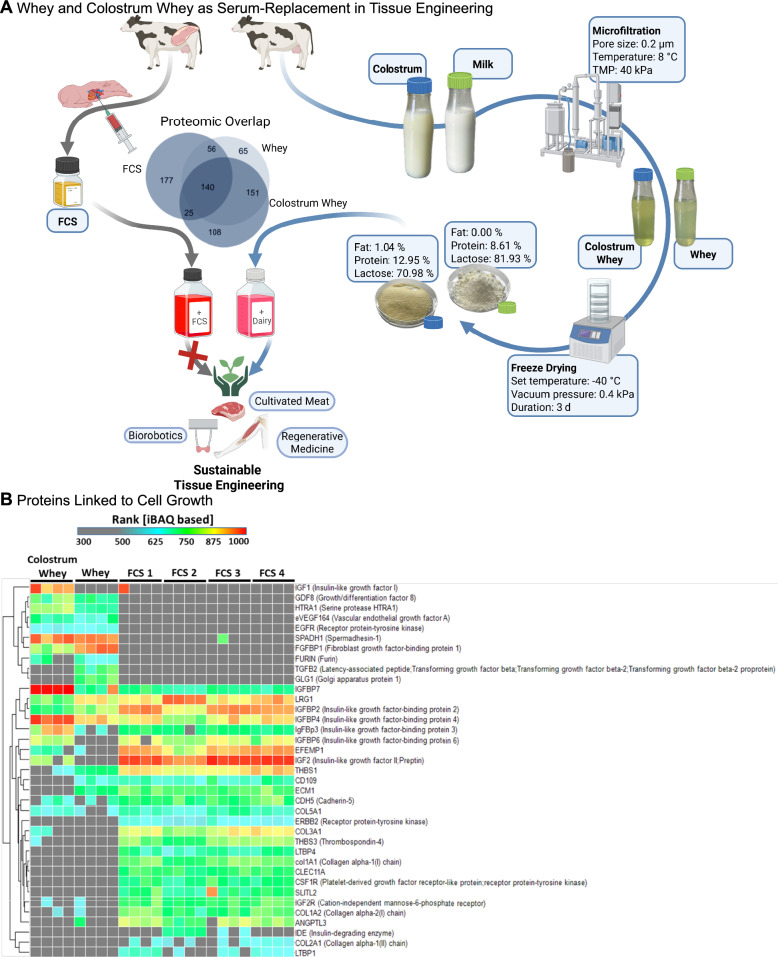
Overview of the project and composition of whey and colostrum whey.** A** Production of whey and colostrum whey as a possible FCS-replacement in skeletal muscle tissue engineering. Measurements of the primary nutritional composition of whey and colostrum whey are indicated. The Venn diagram illustrates overlap between whey, colostrum whey, and FCS in all proteins identified. **B** The heatmap shows hits associated with proliferation in colostrum whey, whey and four different FCS products

The separation of the serum phase from milk and colostrum was conducted in the pilot dairy for research and training at the University of Hohenheim (Fig. 1). The raw milk was obtained from the research station Meiereihof (University of Hohenheim, Stuttgart, Germany), and first separated at 50 °C into cream and skim milk using a disk separator (SA 10, Frautech Separators SRL, Vicenza, Italy). The colostrum kindly provided as an already skimmed product by Colostrum BioTec GmbH (Königsbrunn, Germany).

Both skimmed products were filtered on a batch cross-flow plant (MMS Membrane Systems AG, Zurich, Switzerland) using a 3.8″ spiral wound 0.2 µm membrane (Nadir MV020, Area 5.7 m^2^, Mann + Hummel GmbH, Wiesbaden, Germany). Therefore, a volume of 60 L of each was concentrated at a transmembrane pressure of 40 kPa at 8 °C to a concentration factor of 2. The permeate, containing the whey proteins, was collected and analyzed for protein, lactose, fat, and dry matter content using an FTIR spectrometer (LactoScope FTIR Advance, Delta Instruments, Drachten, Netherlands).

Subsequently, serum phases, in the following referred to as whey and colostrum whey, were aliquoted and stored at −20 °C. For cell culture experiments, whey and colostrum whey were lyophilized and stored at −20 °C.

### Profiling of free amino acids

For profiling of free amino acids, a liquid sample was filtered through a 0.2 µm cellulose acetate filter. Depending on the sample content, it was diluted 1:5 or 1:10 with norvaline (Sigma-Aldrich, St. Louis, Missouri, USA) as an internal standard. Amino acids were derivatized using AccQ-Tag Ultra Derivatization Kit according to the manufacturer's instructions (Waters, Eschborn, Germany).

Analysis was performed on an ACQUITY UPLC H-Class-System equipped with an ACQUITY-UPLC tunable ultraviolet (UV) detector and an ACQUITY QDa mass selective detector (MS; Waters). Separation was achieved using an AccQ Tag™ Ultra C18 column (1.7 µm particle size, 2.1 × 100 mm) (Waters) at 49 °C and gradient elution with mobile phases AccQ Tag Ultra Eluent A, H_2_O/AccQ Tag Ultra Eluent B (90/10), H_2_O and AccQ Tag Ultra Eluent B at a flow rate of 0.7 mL/min. The gradient was set according to the manufacturer's instructions (Waters). Amino acids were detected and quantified at 260 nm. Peaks were identified by retention times and mass spectra compared to reference standards. The MS scan range was *m/z* 200–400 in positive ionization mode. Data was processed with the Empower 3 software (Waters). For quantitation via external calibration, standards with six concentrations (6.25 to 250 µmol/mL) were used.

### Quantification of minerals and trace elements

An 0.5 mL aliquot of liquid sample was diluted 1:10 for ICP-OES measurements (Agilent 5110, Agilent Technologies, Santa Clara, California, USA). Analysis of elements was performed at element-specific wavelengths with calibration curves created from respective standards. All measurements were performed at room temperature.

### Lactate measurements by ion chromatography

Sample were extracted following Chen et al. 2014 with modifications. An aliquot of the liquid sample was centrifuged (15,000 rounds per minute (rpm), 20 °C, 10 min) (Andreas Hettich, Tuttlingen, Germany). The supernatant was filtered using a 0.45 µm membrane filter (CHROMAFIL®Xtra IC-45/25, Macherey–Nagel, Düren, Germany). Dilutions were prepared to ensure measurements fell within calibration ranges. 25 µL of the diluted solution were analyzed using an ion chromatograph Integrion RFIC system, equipped with KOH eluent generator, ACRS 500 suppressor, and conductivity detector (Thermo Fisher Scientific, Dreieich, Germany).

Chromatographic separation of organic acids and anions was performed on a Dionex Ion Pac AS11-HC Analytical, 2 × 250 mm column with a guard column (P/N 052961 and 052963, Thermo Fisher Scientific) at 30 °C column temperature. The flow rate was set to 0.38 mL/min. The mobile phase, generated with a Dionex EGC III KOH Eluent Generator, started at 1 mmol KOH for 15 min and increased to 45 mmol KOH over 15 min. After rinsing for 3 min with 60 mmol KOH, the mobile phase returned to initial conditions. Analytes were detected using a conductivity detector. Lactate quantification was performed using commercial standards (Carl Roth, Karlsruhe, Germany) at five concentrations ranging from 1 to 20 mg/L.

### Quantitative determination of riboflavin

Sample preparation of riboflavin followed previously described methods (Benvenuti et al. 2016) with modifications. Liquid whey samples were filtered using a 0.2 µm polyamide membrane. 990 µL of sample were mixed with 10 µL 0.5 M NaOH, and sonicated (Elma Schmidbauer, Singen, Germany) for 5 min at 30 °C. The mixture was diluted equal parts with mobile phase (A + B, 98 + 2) and 5 µL were analyzed on an UPLC ACQUITY UPLC H-Class System equipped with an ACQUITY-UPLC tunable-UV detector and an ACQUITY QDa mass selective detector (MS) (Waters).

Chromatographic separation of riboflavin was performed on an ACQUITY UPLC-HSS T3, 1.8 µm, 100 × 2.1 mm with guard column (Waters) at 30 °C using mobile phases: A (10 mmol ammonium acetate with 0.1% formic acid in water) and B (10 mmol ammonium acetate in methanol with 0.1% formic acid). The flow rate was set to 0.35 mL/min, and the sample manager temperature was set to 5 °C. The gradient started at 1% B, increased to 55% B over 3 min, then to 98% B for 3 min, before returning to initial conditions. Detection was performed at 267 nm and at 377 m/z (SIR) in positive ionization mode. Quantification used external calibration with commercial standards (Merck, Darmstadt, Germany) at six concentrations, ranging from 0.03 to 1.5 mg/L.

### Mass spectrometry proteomics sample preparation

For mass spectrometry, samples were mixed 1:1 (v/v) with 2% SDS, 150 mM Tris HCl (pH 6.8), and 10 mM DTT. Proteins were initially isolated by methanol/chloroform extraction (Wessel and Flügge 1984). The pellets were dissolved in 2% sodium deoxycholate (SDC) in 100 mM Tris HCl (pH 8.5). The sample was diluted 1:10 (v/v) with 100 mM Tris HCl (pH 8.5) and adjusted to a final protein concentration of 1 µg/µL with 0.2% SDC, 100 mM Tris HCl (pH 8.5). Samples were reduced and alkylated with 10 mM TCEP and 40 mM CAA (20 min, 60 °C, 800 rpm) and further processed according to the Single-pot, solid-phase-enhanced sample preparation (SP3) protocol, as described in Hughes et al. 2019. Briefly, proteins were bound to SP3 magnetic beads (1:1 Mixture of SpeedBeads™ magnetic carboxylate modified particles 50 mg/mL; Cytiva; CAT No: 45152105050250 and 65152105050250) by adding ethanol to a final concentration of 70%, and digested on the beads with trypsin (1:100 w/w, enzyme/protein, Roche, Basel, Switzerland) and LysC (1:200 w/w, enzyme/protein, FUJIFILM Wako Pure Chemical Corporation, Osaka, Japan) in 0.2% SDC, 50 mM ammonium bicarbonate buffer (20 h, 37 °C, 1200 rpm). Formic acid (FA) was added to the samples to a final concentration of 2%. Precipitated SDC was pelleted by centrifugation at 20,000×*g* for 15 min. Supernatants were dried under vacuum and peptides were dissolved in 0.2% FA and 2% Acetonitrile (ACN).

### Proteomics liquid chromatography-mass spectrometry

NanoLC-MS/MS experiments were performed on an Ultimate 3000 nano-RSLC coupled to an Exploris 480 mass spectrometer via a Nanospray-Flex ion source (all Thermo Fisher Scientific). Peptides were concentrated and desalted on the µPAC Trapping column nano (PharmaFluidics, Ghent, Belgium) and then separated on a 50 cm µPAC nanoLC column (PharmaFluidics) maintained at 45 °C. Peptides were separated using a gradient with the following profile: 2% (700 nL/min)–10% (700 nL/min) solvent B in 4 min, 10 (700 nL/min)–12% (300 nL/min) solvent B in 6 min, 12–22% (300 nL/min) solvent B in 23 min, 22–40% (300 nL/min) solvent B in 9 min, 40–95% (300 nL/min) solvent B in 2 min, isocratic 95% (300 nL/min) solvent B for 2 min, 95% (300 nL/min)–2% (700 nL/min) solvent B in 4 min and isocratic 2% (700 nL/min) solvent B for 10 min. The solvents used were 0.1% FA (solvent A) and 0.1% FA in ACN/H2O (80/20, v/v, solvent B). MS spectra (m/z = 300–1400) were detected in the Orbitrap at a resolution of 120,000 (m/z = 200). The maximum injection time (MIT) was set to 50 ms and the automatic gain control (AGC) value was set to 3 × 10^6^. Internal calibration of the Orbitrap analyzer was performed using lock-mass ions from ambient air, as described in Olsen et al. 2005. The MS was operating in the data-dependent mode, selecting the 15 most abundant peptide precursor signals for fragmentation (HCD, normalized collision energy of 30%). For MS/MS analysis, only precursor charge states from 2 to 4 were considered. The monoisotopic precursor selection was set to peptides, and the minimum intensity threshold was set to 1 × 10^5^. MS/MS scans were performed in the Orbitrap with a resolution of 15,000 and an isolation width of 1.5 Da. The AGC target was set to 9 × 10^4^, a max injection time was set to 70 ms, and the first mass was set to 120 m/z. Dynamic exclusion was set to 60 s with a tolerance of 8 parts per million (ppm).

### Mass spectrometry data analysis

Raw files were imported into MaxQuant (Cox and Mann 2008) version 2.0.1.0 for protein identification and intensity-based absolute quantification (iBAQ) of proteins. Protein identification in MaxQuant was performed using the database search engine Andromeda (Cox et al. 2011). MS spectra were searched against the *Bos taurus* (UniProt, Taxon ID 9913; Jan. 2022) sequence database (UniProt: a worldwide hub of protein knowledge 2019). Reversed sequences as decoy database and common contaminant sequences were added automatically by MaxQuant. Mass tolerances of 4.5 ppm for MS spectra and 20 ppm for MS/MS spectra were used. Trypsin was specified as the enzyme and two missed cleavages were allowed. Carbamidomethylation of cysteines was set as a fixed modification and protein N-terminal acetylation and oxidation were allowed as variable modifications. The ‘match between runs’ feature of MaxQuant was enabled with a match time window of 1 min and an alignment time window of 20 min. Peptide and protein false discovery rate thresholds were set to 0.01.

The MaxQuant output file (protein groups table) was loaded into Perseus version 1.6.14.0 (Tyanova et al. 2016). Protein groups with reverse sequence matches and known contaminants that were not assigned to the *Bos taurus* database were eliminated. iBAQ values from MaxQuant were log_2_-transformed and filtered for a minimum of two valid values in at least one experimental group (N = 4 replicates). GO-Terms (GOMF names) were linked to the identified protein groups in the dataset. After filtering, the data matrix contained 1008 entries that were used for a rank-based normalisation strategy. Therefore, in each LC–MS/MS run, rank numbers were assigned to the iBAQ values top down (the highest iBAQ value resulted in the highest rank number). All entries of a LC–MS/MS run that lacked an iBAQ value (non-valid values) were assigned to the same lowest possible rank number within the individual LC–MS/MS run. The GO-Term annotations were used to isolate protein groups that were functionally linked to at least one cell growth associated GO-Term (GOMF name: “epidermal growth factor binding”, “insulin-like growth factor binding”, “epidermal growth factor”, “receptor activity”, “insulin-like growth factor I binding”, “epidermal growth factor receptor binding”, “insulin-like growth factor II binding”, “growth factor activity”, “insulin-like growth factor receptor binding”, “growth factor binding”, “transforming growth factor beta binding”, “growth factor receptor binding”, “transforming growth factor beta receptor activity”, “growth hormone receptor activity”, “transforming growth factor beta receptor binding”, “insulin binding”, “transforming growth factor beta receptor activity, type III”, “insulin receptor binding”). The isolated data was used as input for the Euclidean distance hierarchical cluster analysis. For the Venn diagram comparison, the iBAQ quantifiable protein groups were used as input.

### Cell culture

C2C12 cells (ACC 565, DSMZ, Braunschweig, Germany) were maintained in DMEM high glucose (L0101, Biowest, Nuaillé, France) supplemented with 1% stable glutamine (X0551, Biowest) and 10% FCS (P30-3306, PAN-Biotech, Aidenbach, Germany) at 37 °C and 5% CO_2_ in T-flasks (Greiner Bio-One, Frickenhausen, Germany). Cells were passaged with 0.05% trypsin (11570626, Fisher Scientific, Schwerte, Germany) every two to three days, keeping confluency under 70%.

### Short-term proliferation assay

For the dairy-based media, lyophilized whey or colostrum whey were dissolved at concentrations of 5 mg/mL and 2.5 mg/mL in DMEM high glucose with 1% stable glutamine, RPMI 1640 (L0498, Biowest), or MCDB 131 (10372019, Thermo Fisher Scientific, Waltham, USA). Dissolution was performed at 37 °C with frequent vortexing at the lowest speed. The media were then filtered through a 0.2 µm PES-filter (15206869, Fisher Scientific, Schwerte, Germany) and 100 U/mL penicillin/streptomycin (P06-07100, PAN-Biotech) was added. For each base medium, a positive control with 10% FCS and a negative control without any supplementation were prepared.

Cells were seeded in proliferation medium at 3000 cells per well into 96-well plates (655180, Greiner Bio-One) and allowed to adhere for 6 h. The medium was then replaced with the tested conditions. Medium exchange was done after 48 h. After another 48 h, cell numbers were determined by DNA-quantification.

DNA-quantification was performed as described in Ligasová and Koberna 2019 with minor modifications. To prepare a standard curve, cells were seeded into 96-well plates at densities ranging from 1000 to 80,000 cells per well and allowed to adhere for 3 h. After washing with 100 µL of phosphate-buffered saline (PBS) (PBS-2A, Capricorn Scientific, Ebsdorfergrund, Germany) and cells were fixed with 70% ethanol (5054.1, Carl Roth) for 10 min. The Ethanol was discarded and the plates were left at room temperature until remaining ethanol was evaporated entirely. DNA was stained with 3 µM 4′,6-Diamidino-2-phenylindol (DAPI) (18860.01, SERVA Electrophoresis, Heidelberg, Germany) in 20 mM TRIS (9090.6, Carl Roth) with 150 mM NaCl (6307-1KG, Th. Geyer, Renningen, Germany) (pH 7) for 30 min at 168 rpm. Wells were washed three times (5 min, 168 rpm) with a washing solution containing 2 mM CuSO_4_ (1027900250, Sigma-Aldrich), 500 mM NaCl, 20 mM sodium citrate (1064480500, Sigma-Aldrich), and 0.2% Tween20 (P9416-100ML, Sigma-Aldrich) at pH 5. The pH was readjusted with 20 mM TRIS and 150 mM NaCl (pH 7) for 5 min. Bound DAPI was eluted using 150 µL 20 mM TRIS with 2% sodiumdodecylsulfate (20771.01, SERVA Electrophoresis) for 15 min at 168 rpm. Finally, 100 µL eluate was transferred to black 96-well plates (655076, Greiner Bio-One) and the fluorescent signal was measured at 370 nm excitation and 460 nm emission. Cell numbers were calculated from the standard curve.

To investigate the morphology of the cells, 9000 cells were seeded per well in an 8-well µ-slide (80826, ibidi, Gräfeling, Germany) and treated as described above. On day 4, cells were fixed using 4% Roti Histofix (P087.3, Carl Roth) for 10 min and permeabilized for 5 min using 0.1% Triton X-100 (T8787-100, Sigma-Aldrich) in PBS. Actin was stained using the ReadyProbes™ Reagent F-Actin Phalloidin Conjugates (R37112, Thermo Fisher Scientific). Two drops of the reagent were used in 1 mL PBS and 1:1000 DAPI, and cells were incubated for 30 min. After three washes with PBS, images were acquired using a fluorescence microscope (Axio Observer) with an Axiocam 305 camera and ZEN blue software (Carl Zeiss AG, Jena, Germany).

### Long-term culture in colostrum whey media

For the long-term evaluation of a colostrum whey (CW) based medium, C2C12 cells were cultured in RPMI 1640 with 1% penicillin/streptomycin and five different supplementations: 5 mg/mL CW, 1% ITS (ITS-H, Capricorn Scientific), 5 mg/mL CW + 1% ITS, 10% FCS and no supplementation. Cells were seeded on collagen I (354236, Corning, New York, USA) coated T-flasks and passaged every two to three days with accutase (P10-21500, PAN-Biotech), avoiding confluences over 70%. Doubling times for each passage were calculated from the cell counts. Doubling times from day 7 to day 30 were used to calculate the mean doubling times. After 30 days of culture, 40,000 cells were seeded into an 8-well µ-slide and fixed after 6 h. Actin staining was used to identify potential changes in cell morphology as described before.

### Myogenic differentiation

After 30 days of culture, cells were seeded at 1 × 10^6^ cells per well in 6-well plates (657160, Greiner Bio-One) for RNA isolation and 100,000 cells per well in 48-well plates (677180, Greiner Bio-One) for immunostaining and left to adhere for 6 h. Undifferentiated control cells were either fixed for 10 min with 4% Roti Histofix (48-well plates) or harvested for RNA isolation (6-well plates). The remaining cells were differentiated by switching to differentiation medium. Differentiation medium consisted of DMEM/F12 (L0092, Biowest) with 1% ITS and 10 µM SB431542 (13031, Cayman Chemical, Ann Arbor, Michigan, USA). Medium was exchanged after 72 h. Cells were fixed or harvested after an additional 48 h. Differentiation was evaluated by immunofluorescence staining and quantitative polymerase chain reaction (qPCR).

### Immunofluorescence staining

Cells were fixed as described for actin staining, then washed twice with PBS and stored in PBS at 4 °C until further use.

Cells were permeabilized with 0.1% Triton X-100 in PBS for 15 min at room temperature. Blocking was performed with 1% bovine serum albumin (BSA, 01400.100, Biomol, Hamburg, Germany) and 0.1% Tween20 in PBS for 1 h at room temperature. Subsequently, samples were incubated overnight at 4 °C with anti-MYH4 mouse antibody (14-6503-82, Thermo Fisher Scientific) diluted 1:500 in blocking solution. On the next day, samples were washed three times for 10 min with 0.1% Tween20 in PBS. Primary antibody was detected with anti-mouse goat antibody Cy3 (115-165-003, Jackson ImmunoResearch, Ely, UK) diluted 1:250 in blocking solution for 1 h at room temperature. Cells were washed three times and nuclei were stained with 0.1% DAPI for 10 min. After washing with PBS, the samples were stored at 4 °C or used directly for analysis. Images were acquired as described for actin staining.

### Quantitative polymerase chain reaction

RNA was isolated with the NucleoSpin RNA/Protein Kit (740933.50, Macherey–Nagel, Düren, Germany) according to the manufacturer’s instructions with modifications. Cells were harvested by lysis with 350 µL of RP1 buffer and 20 mM TCEP (740395.107, Macherey–Nagel), then stored in liquid nitrogen until isolation. The lysate was filtered through a NucleoSpin Filter at 11,000 × g for 1 min. Subsequently, the filtrate was mixed with 350 µL of 70% EtOH, loaded on the membrane and centrifuged at 11,000*×*g for 30 s. The membrane was washed with 350 µL of MDB (11,000×*g*, 1 min) and genomic DNA was digested with 95 µL rDNase reaction mixture for 15 min at room temperature. The membrane was washed with 200 µL RA2 (11,000×*g*, 30 s), 600 µL RA3 (11,000×*g*, 30 s), and 250 µL RA3 (11,000×*g*, 2 min). Finally, the RNA was eluted with 60 µL of RNase-free water and its concentration was measured using the NeoDot microvolume spectrophotometer (NB-12-5001, Biotrend, Cologne, Germany). RNA was stored at −80 °C.

Biozym cDNA Synthesis kit (331470X, Biozym Scientific, Hessisch Oldendorf, Germany) was used for cDNA synthesis according to the manufacturer’s instructions. The reaction mix was prepared as shown in Table 1. The reaction mix was incubated at 50 °C for 30 min and at 99 °C for 5 min. The cDNA was stored at −20 °C until further use.

**Table 1:** Reaction mix for cDNA synthesis

Component	Volume
dNTP Mix	2 µL
RNase Inhibitor	0.5 µL
Oligo (dT) Primer	0.5 µL
5 × cDNA Synthesis Buffer	4 µL
Reverse Transcriptase	1 µL
RNA Template	for 1 µg of RNA
PCR Grade Water	for 20 µL reaction volume

Primers (Eurofins Scientific, Luxembourg City, Luxembourg) for qPCR were designed with PrimerBlast and are shown in Table 2.

**Table 2:** Primer pairs used for quantification of myogenic differentiation

Gene	Forward primer (5′–3′)	Reverse primer (5′–3′)
*B2m*	ATCCAAATGCTGAAGAACGGG	GCAGGCGTATGTATCAGTCTC
*Myh1*	AAGGGTCTACGCAAACACGA	TGCGGAATTTGGAGAGGTTGA
*Myh2*	TCATAAGCGAAGAGTAAGGCTG	CAGGATGGAATAGAATCACACAGG
*Myh4*	CCGAGAGGTTCACACTAAAGTC	TACAGGACAGTGACAAAGAACG
*Myog*	GGATATGTCTGTTGCCTTCCC	GACAGCCCCACTTAAAAGCC

For each gene in qPCR, a mastermix of 10 µL 2× qPCR S’Green BlueMix (331416XL, Biozym Scientific) and 0.6 µL each of forward and reverse primer (10 µM) was prepared. Template cDNA was diluted with PCR-grade water to 5 ng per 8.8 µL. 11.2 µL of master mix and 8.8 µL of diluted cDNA were mixed for each reaction in a 96-well PCR plate (710876, Biozym Scientific). No reverse transcriptase and no template controls were included. Samples and measurements were only used if quality control by the negative controls was passed. Measurement was performed on a LineGene 9000 (Biozym Scientific). Initial denaturation was performed at 95 °C for 2 min. A program of denaturation at 95 °C for 15 s, annealing at 57 °C for 15 s, and elongation at 72 °C for 20 s was run for 40 cycles. The data obtained were normalized using *B2m* as the housekeeping gene, according to Pfaffl 2001.

### Cryopreservation of C2C12 cells

The freezing mix with CW (FMC) was prepared by dissolving 10 mg CW per mL in RPMI 1640 and filtration as described before. For the FCS-based freezing mix, 20% FCS were mixed with RPMI and 10% DMSO (10103483, Fisher Scientific) was added to both freezing mixes. Cells adapted to each culture medium were resuspended at 0.5 × 10^6^ cells/mL and frozen in 1 mL aliquots at a rate of approximately 2 °C/min down to −80 °C. The next day, cells were transferred to liquid nitrogen for long-term storage. After 3 months, cells were thawed quickly in a water bath at 37 °C. Cells were resuspended and a 20 µL aliquot was taken to assess viability with the CellDrop AO/PI Viability Assay (31CD-AO-PI-1.5, Biozym Scientific) by mixing it 1:1 and counting live and dead cells with the Celldrop FL cell counter (Biozym Scientific). The rest of the cell suspension was washed immediately after thawing with 9 mL of culture medium, seeded, and cultured as described before. In the second passage after thawing, cell counts were assessed to calculate the doubling time to this passage.

For cell cycle analysis, cells from the second passage after thawing were aliquoted to 550,000 cells in 1.5 mL reaction tubes (616201, Greiner Bio-One) and centrifuged at 200*×*g for 5 min. After resuspension in 100 µL PBS without Ca^2^⁺/Mg^2^⁺ (PBS-1A, Capricorn Scientific), 1 mL of ice-cold 70% ethanol was added dropwise, while vortexing on the slowest setting. Fixation continued for 2 h at −20 °C. Subsequently, cells were washed twice with FACS buffer, consisting of PBS without Ca^2+^/Mg^2+^ with 0.5% BSA and 2 mM EDTA (11568896, Fisher Scientific). Primary staining was performed by resuspension in 100 µL of a 1:50 dilution of anti-Ki67 antibody (ab16667, Abcam, Cambridge, UK) in FACS-buffer and incubation for 30 min at room temperature. Cells were washed by addition of 1 mL FACS buffer. Secondary staining was performed with a 1:50 dilution of the anti-rabbit CruzFluor™ 647 antibody (sc-516251, Santa Cruz Biotechnology, Dallas, Texas, USA). After washing, DNA was stained with a solution of 1:500 DAPI in FACS-buffer for 30 min at room temperature. Analysis was performed on the MACSQuant Analyzer 10 Flow Cytometer (Miltenyi Biotec, Bergisch Gladbach, Germany). Gating strategy is illustrated in Supplementary Fig. 1. After identification of single cells, G0/G1-, S-, and G2/M-Phase were differentiated by the fluorescence intensity of DAPI, which is associated with single to double DNA-content. Cells in G0- and G1-phase were differentiated as Ki67 negative and positive cells of the G0/G1 gated cells.

### Static 3D suspension culture

3D suspension culture was performed in 96-well U-bottom plates (650,185, Greiner Bio-One) coated with anti-adherence solution (7010, StemCell Technologies, Vancouver, Canada). Cells adapted to the culture medium for 9 days were seeded at densities of 5,000, 10,000, and 20,000 cells per well in the respective medium. Culture media was exchanged on days 2, 4, 7, 9 and 11. Spheroids were transferred to new culture plates on day 7. Analysis of viability and size, as well as harvesting for DNA-quantification, was carried out on days 1, 3, 7, and 14 after cell seeding. Spheroid size was evaluated by measurement of the spheroid area in phase contrast images. Viability was assessed using the Live/Dead Viability/Cytotoxicity kit (10237012, Fisher Scientific). 2 µL of ethidium homodimer-1 and 0.5 µL of calcein AM were used per mL of PBS and added to the spheroids after aspiration of the media. Pictures were acquired after 30 min of staining at room temperature. Spheroids for DNA-quantification were fixed by exchanging the media with ice-cold 70% ethanol and stored at −20 °C until further analysis. The Assay was adapted from DNA-quantification in 2D as described above. Incubation times were prolonged to 1 h for DAPI staining, 40 min for washing, and 1 h for elution to account for longer diffusion times. For all three analyses, at least three spheroids were measured as technical replicates for each condition and experiment.

### Statistical analysis

Data from chemical analyses were gained from two measurements and data from mass-spectrometry from four measurements. Values were depicted as mean ± 1st standard deviation (SD).

Data from the short-term proliferation assay were obtained from six, while all other cell culture data were from three independent experiments. The Gaussian distribution was tested for by the Shapiro–Wilk test. In case of normal distribution, values were expressed as mean ± 1st SD, otherwise in geometric mean ×/÷ geometric SD factor. Significances were determined by two-way analysis of variances (ANOVA) followed by Dunnett multiple comparisons test for short-term proliferation assay and ANOVA followed by Šídák for qPCR relative gene expression data. Significances for other cell culture data were determined using unpaired t-test or Mann–Whitney test, depending on the distribution type. Statistical testing was performed with GraphPad Prism 10.4.1. Results were considered statistically significant with *p ≤ 0.033; **p ≤ 0.002; ***p ≤ 0.001.

## Results

### Whey and colostrum whey contain possible pro-proliferative components

Initially, whey was generated by microfiltration of colostrum and milk (Fig. 1). Both products were analysed for their composition. With a dry mass of 4.76%, whey (W) was higher in content than colostrum whey (CW; dry mass: 1.93%). Analysis of the dry mass revealed that the difference was primarily due to lactose, accounting for 81.93% of the dry mass for W and 70.98% for CW. CW had a higher content of protein (12.95% versus 8.61%), residual fat (1.04% versus 0%), and other components (15.03% versus 9.46%) compared to W. Further analysis revealed a broad spectrum of free amino acids present in both, CW and W (Supplementary Table 1). However, concentrations were negligibly low compared to base cell culture media, such as DMEM (Supplementary Table 2). Concentrations of trace elements were below detection limits, while overall mineral content was low enough not to disturb osmolarity. Lactate and riboflavin were also found at comparably low concentrations.

Whey proteins are one of the main components of whey and colostrum whey and can be a source of bioactive proteins. To gain further insights into the protein composition, CW, W, and FCS samples were subjected to NanoLC-MS/MS analysis. The Venn diagram in Fig. 1A and summarizes the overlap between the identified proteins of the three substances. Variances between the analysed FCS products were negligible (Supplementary Fig. 2). While CW and W share the most considerable overlap with 291 common proteins, W and FCS only share 196 proteins, followed by CW and FCS with 165. Furthermore, the highest amount of unique hits (177) was found for FCS. CW and W had 108 and 65 unique hits, respectively. A closer look into proteins connected to cell growth-associated GO-Terms revealed a spectrum of proliferation-modulating proteins (Fig. 1B). Among those were proteins involved in the signalling pathways of IGF (IGF-1, IGF-2, IGFBP2 (IGF binding protein 2), IGFBP3, IGFBP4, IGFBP6, IGFBP7, IGF-2 receptor) and TGF-β (TGF-β2, HTRA1, LTBP4 (latent TGF-β binding protein 4), LTBP1, leucine-rich alpha-2-glycoprotein 1 (LRG1), GDF8 (growth/differentiation factor 8). While proteins involved in IGF-signalling were most frequently present in FCS and CW, proteins modulating TGF-β-signalling were common in all three substances. IGF-1 was present exclusively in CW, IGF-2 in CW and FCS, and TGF-β2 in W. Several forms of IGFBPs were detected in different patterns in the samples. Furthermore, myostatin (GDF8) and serin-protease HTRA1 were found in CW and W. Overall, most of the overlap in the proliferation-modulating proteins between CW, W, and FCS consisted of IGFBPs. Variations in the proliferation-modulating proteins between FCS products were minor, with FCS 2 being the only one with slight differences in leucine-rich alpha-2-glycoprotein 1 (LRG1), IGFBPs, EGF-containing fibulin-like extracellular matrix protein 1 (EFEMP1), and insulin-degrading enzyme (IDE).

### Colostrum whey supports short-term proliferation

**Fig. 2:**
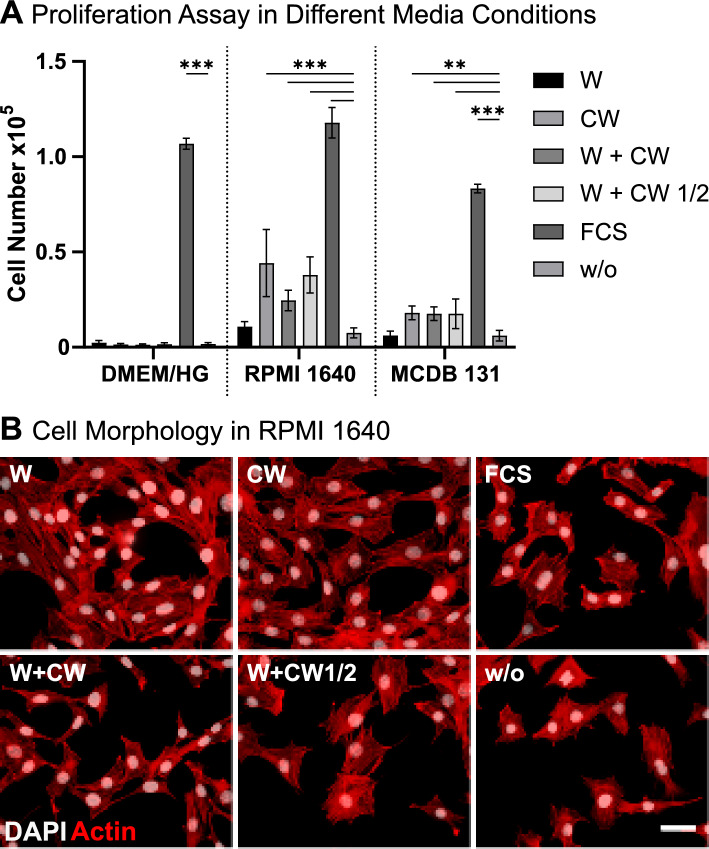
Short-term culture of C2C12 under different supplementations in DMEM high glucose, RPMI 1640, and MCDB 131. **A** Cell numbers measured after 4 days of culture under supplementation with whey (W), colostrum whey (CW), their combinations at 5 mg/mL or 2.5 mg/mL each, 10% FCS (FCS), and without supplementation (w/o). **B** Cell morphology after 4 days in RPMI 1640 with different supplementations. *White*: DAPI; *Red*: Actin; Scale bar: 50 µm. **p ≤ 0.002; ***p ≤ 0.001. n = 6

As a first evaluation of possible pro-proliferative effects of the two dairy-based byproducts, C2C12 cells were cultured under supplementation with W, CW, and their combinations. Growth was analysed in comparison to supplementation with 10% FCS and a negative control without any supplementation. While DMEM high glucose is the standard growth medium of C2C12 cells, two other media applicable to a variety of cells, namely RPMI 1640 and MCDB 131, were also used as base media to see whether a possible effect might be dependent on the base medium. Composition of these media is provided in Supplementary Table 2. Over 4 days of culture, DMEM showed no positive effects on proliferation from W, CW, or their combinations compared to the negative control (Fig. 2A, all cell numbers in Supplementary Table 3). However, the other two base media significantly supported cell growth when using CW alone or in combination with whey. Supplementation with W did not have significant effects on cell growth. Overall, CW in RPMI 1640 yielded the highest short-term proliferation, albeit the cell number being 2.7 times lower than in 10% FCS. After 4 days of culture, cells did not show any prominent morphological differences between dairy supplementation and FCS (Fig. 2B). As lactose was the main component of the produced whey fraction, its sole supplementation was tested to ensure that the pro-proliferative effect of CW was not only due to a higher carbon and energy supply by lactose. This single supplementation did not enhance proliferation compared to the negative control (Supplementary Fig. 3).

### Colostrum whey medium (CM) allows for 30 days of culture

**Fig. 3:**
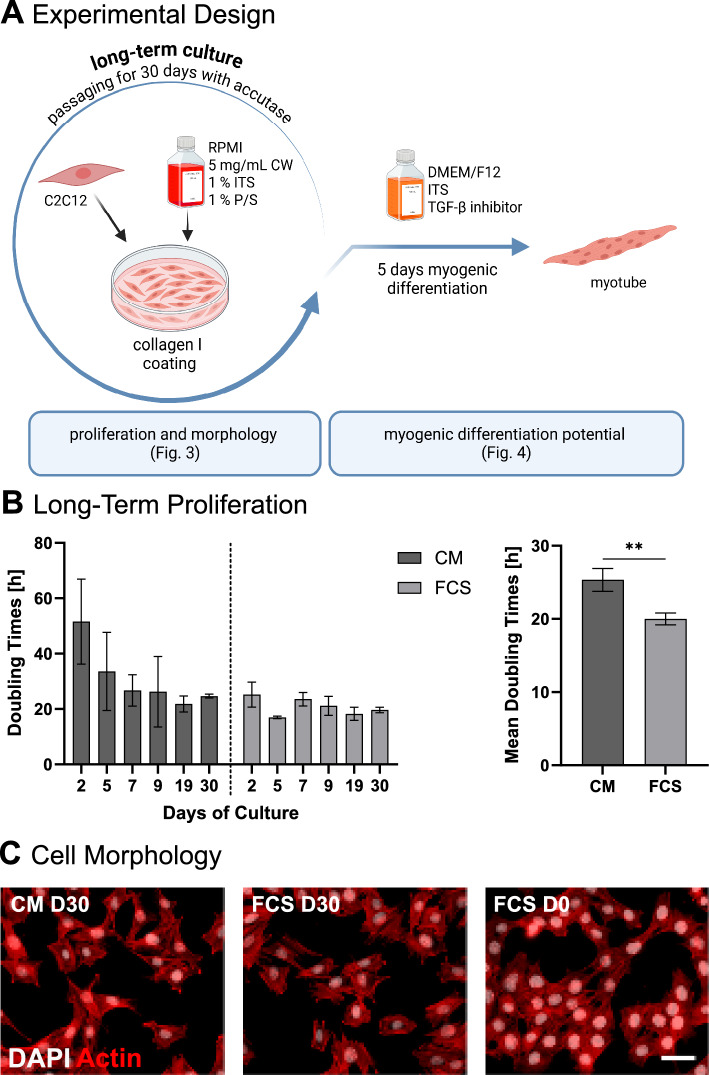
Long-term culture of C2C12 in CM and FCS-containing media. **A** Experimental design of long-term culture and following myogenic differentiation. **B** Doubling times at passages on indicated days. The right graph shows the mean doubling times calculated from the passages between day 7 and 30. **C** Cell morphology after 30 days of culture in CM or FCS-containing medium and in control (FCS). *White*: DAPI; *Red*: Actin; Scale bar: 50 µm. ** *p* ≤ 0.002. n = 3

To test whether CW-supplemented RPMI medium can sustain long-term culture of C2C12 cells, cells were cultured for 30 days under different conditions. Besides the single supplementation with CW, ITS (Insulin Transferrin Selenium) was also added to the tested conditions, as it is a common supplement for serum-free culture. Problems with low cell numbers during passaging and reattachment were addressed by using accutase instead of trypsin and coating the culture surfaces with collagen I. While cells cultured without supplementation or only with ITS died off during the first 5 days, cells in CW were viable until between day 7 and 12 (Supplementary Table 4). Cells in RPMI 1640 supplemented with CW and ITS (Colostrum Medium/CM) adapted to serum-free culture until day 7 and then exhibited stable growth until day 30 (Fig. 3B). The mean doubling time in CM of about 25.33 ± 1.57 h is in a suitable range for cell culture, albeit being slightly higher than in FCS with about 20.00 ± 0.80 h. At the end of the 30-day culture period, no morphological changes were observed between the two media types or compared to a day 0 control (Fig. 3C).

### Cells in CM maintain myogenic potential

**Fig. 4:**
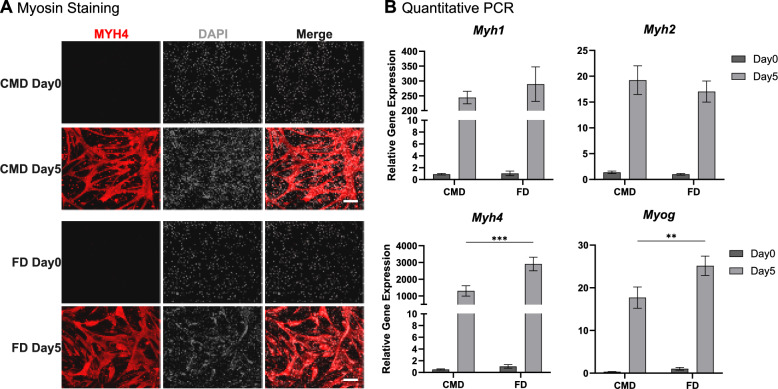
Evaluation of the myogenic potential after 30 days of culture in CM or FCS-containing medium. **A** Staining of myotubes by immunofluorescence detection of MYH4 after 0 and 5 days in differentiation medium (differentiation after CM: CMD; differentiation after FCS-containing medium: FD). *White*: DAPI; *Red*: MYH4; Scale bar: 200 µm. **B** Relative fold change in gene expression of *Myh1*, *Myh2*, *Myh4,* and *Myog* compared to FD day 0. **p ≤ 0.002; ***p ≤ 0.001. n = 3

Since the ability to differentiate is crucial for using C2C12 cells in tissue engineering, their potential for myogenic differentiation was assessed after 30 days of culture in CM. Cells were induced to differentiate in serum-free differentiation medium for 5 days and analysed by immunofluorescence staining of myosin heavy chain 4 (MYH4; Fig. 4A). Regardless of the used proliferation medium, cells demonstrated high levels of myogenic differentiation, forming long networks of multinucleated myotubes with strong MYH4 expression. However, in the approach of differentiation after CM adaptation (CMD), some MYH4-positive cells appear aligned but not fused yet, while some smaller myotubes seem to detach or die. Additionally, myotubes appear thinner compared to their counterparts differentiated after 10% FCS (FD). This size difference is more evident when quantifying MYH4 signals, which shows slightly lower myotube area per picture (0.65 ± 0.04 mm^2^ to 0.81 ± 0.04 mm^2^; Supplementary Fig. 4). Quantification of myogenic gene expression relative to FD Day 0 (Fig. 4B) also indicated reduced expression of *Myh4* and *Myog* in CMD (1303.00 ± 308.60 and 17.70 ± 2.50) compared to FD (2909.00 ± 405.80 and 25.16 ± 2.26). Notably, the baseline expression of both genes was slightly lower on Day 0 of CMD (0.51 ± 0.11 and 0.34 ± 0.03) than in FD (1.03 ± 0.30 and 1.04 ± 0.32). CMD and FD exhibited no significant difference in *Myh1* and *Myh2* expression. Both showed the same pattern of expression with high expression of *Myh4*, medium expression of *Myh1* (CMD: 244.00 ± 21.06; FD: 289.20 ± 58.05), and low expression of *Myh2* (CMD: 19.25 ± 2.78; FD: 17.03 ± 2.03). Overall, cells expanded in CM can effectively differentiate into myotubes with a pattern similar to serum-proliferated cells, but with a somewhat reduced differentiation capacity.

### Cryopreservation of cells using colostrum whey

**Fig. 5:**
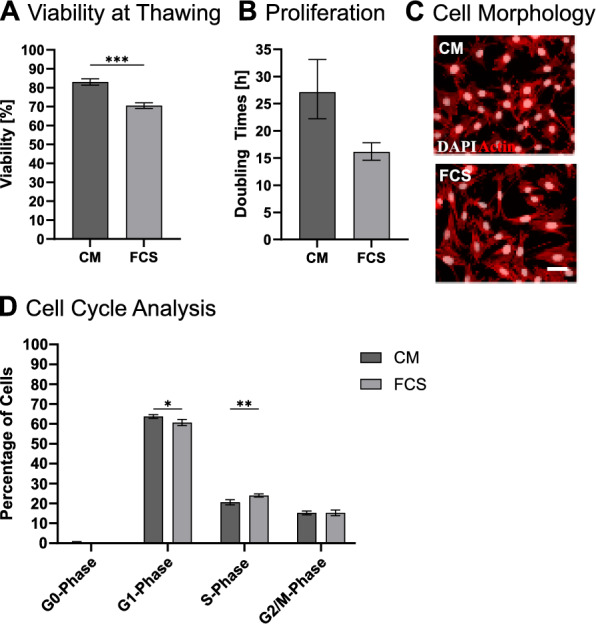
Culture of cells after cryopreservation in CM or FCS-based freezing mixes. **A** Viability of cells directly after thawing. **B** Calculated doubling times by cell number at the second passage after thawing. **C** Cell Morphology at the second passage after thawing. *White*: DAPI; *Red*: Actin; Scale bar: 50 µm. **D** Cell cycle analysis by FACS at the second passage after thawing. *p ≤ 0.033; **p ≤ 0.002; ***p ≤ 0.001. n = 3

As cell cryopreservation is an essential part of cell culture, the suitability of CW used in a freezing mix was evaluated. The freezing mix contained, similar to the serum-containing one with 20% FCS (Freezing Mix FCS: FMF), 10 mg/mL of CW together with 10% DMSO (Freezing Mix Colostrum: FMC). For this experiment, cells were adapted to the culture conditions as shown in the long-term culture experiment, frozen and thawed again. Viability directly after thawing was significantly higher in FMC than in FMF, at 83.06 ± 1.66 and 70.57 ± 1.50 %, respectively (Fig. 5B). The geometric means of the assessed doubling times in passage 2 after thawing were 27.12 ×/÷ 1.22 h for FMC/CM and 16.13 ×/÷ 1.10 h for FMF/FCS (Fig. 5C), which are about the range found in the long-term culture experiment. Cellular Morphology in this passage showed no major changes (Fig. 5D). Furthermore, assessment of the cell cycle revealed small, but significant changes between CM and serum-containing media in G1- and S-phase (Fig. 5E). While G1-phase was elevated in CM (CM: 63.76 ± 0.95; FCS: 60.74 ± 1.54), S-phase was lowered (CM: 20.62 ± 1.29; FCS: 23.97 ± 0.79), indicating that cells in CM take longer to recover from division before entering the next round of DNA duplication. G0- (CM: 0.38 ± 0.39; FCS: 0.06 ± 0.06) and G2/M-phase (CM: 15.22 ± 1.00; FCS: 15.23 ± 1.44) were not significantly affected.

### Myospheres proliferate in CM

**Fig. 6:**
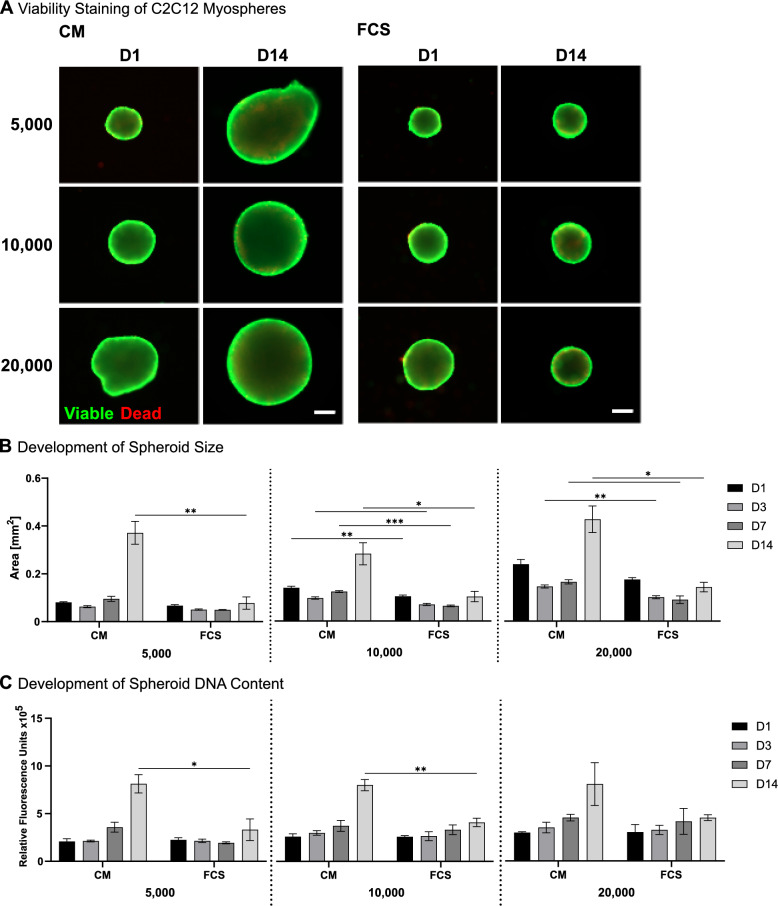
Evaluation of CM in static spheroid culture over 14 days. **A** Viability staining of spheroids in CM and FCS-containing medium on day 1 and day 14. The three different spheroid sizes are indicated by the initial cell numbers of 5000, 10,000, and 20,000 cells per spheroid. *Green*: Viable; *Red*: Dead; Scale bar: 200 µm. **B** Development of spheroid size measured by area in phase contrast microscopy. **C** Changes in spheroid DNA content. *p ≤ 0.033; **p ≤ 0.002; ***p ≤ 0.001. n = 3

After establishing 2D-culture and cryopreservation of C2C12 cells with CM, the next step was to evaluate its use in 3D suspension culture. 3D and suspension culture methods are essential for tissue engineering applications like tissue modelling and scale-up of production processes. For this purpose, CM-adapted cells were cultured as spheroids, called myospheres, in 96-well plate suspension culture for 14 days at seeding numbers of 5000, 10,000, and 20,000 cells. Viability was assessed using live/dead staining, which showed that spheroids remained viable throughout all conditions over the 14-day culture period with only small regions of dead cells at the spheroid core (Fig. 6A; Supplementary Fig. 5). Overall, spheroids displayed typical development of spheroid formation, initially increasing in roundness and compaction, followed by growth. To determine the spheroid size, their area in phase contrast images was measured (Fig. 6B; Supplementary Table 5). Surprisingly, culturing spheroids in CM caused a substantial increase in size over time, with markedly larger spheroids on day 14, regardless of the initial cell number. While their counterparts in FCS-containing medium increased only by a factor of 1.5 between days 7 and 14, they doubled to almost quadrupled their mean size. To verify that the increase in size was not primarily due to extracellular matrix (ECM) production but also to an increase in the overall cell number, the DNA content of individual spheroids was measured (Fig. 6C; Supplementary Table 6). Measurements revealed that spheroids accumulated DNA throughout the culture period. On day 14, the measured DAPI fluorescence for spheroids of the initial size of 5000 and 10,000 cells in CM was significantly higher than in FCS-containing medium (5000: 2.45-fold; 10,000: 1.96-fold). Throughout both measurements, of size and DNA content, myospheres in CM grew with all initial cell seeding densities but reached similar levels of size and DNA content.

## Discussion

Present and future problems like resource scarcity, climate change, and ethics regarding animal welfare call for more sustainable methods and technologies even in seemingly unrelated fields such as tissue engineering. One part of the solution is serum-free culture of cells, which is important not only for biomedical applications, but more so for cultivated meat, since the nature of FCS production directly conflicts with its core idea. This study introduces a straightforward serum substitute for myoblasts utilizing byproducts from the dairy industry, such as whey from raw milk and colostrum. The wheys were analyzed for their chemical composition and tested on C2C12 myoblasts for enhancing proliferation, maintaining their myogenic potential, and supporting a scalable 3D suspension culture.

Wheys can be produced by microfiltration, which separates caseins and fat from whey proteins and other soluble components. About 80% of proteins, fats, and a part of minerals remain in the retentate during this process (France et al. 2024). The result is a whey with a dry mass consisting mainly of lactose, bioactive proteins, amino acids, and various soluble minerals. Colostrum whey has been found to have higher amounts of protein, a broader spectrum of essential and non-essential amino acids, and adequate concentrations of minerals, lactate, and riboflavin compared to whey from raw milk. Taking processing by microfiltration into account, the measurements are consistent with published data on milk and colostrum. Composition analysis shows high similarities with the composition of colostrum in later stages of transition to regular milk at about 5 days after birth (Tsioulpas et al. 2007; Godden et al. 2019; Li et al. 2020a). This suggests that colostrum and even transition milk may represent usable resources, potentially expanding the range and availability of the medium supplement. Despite the broad array of amino acids and minerals of colostrum whey measured in this study and described in literature for colostrum, concentrations are low compared to the abundance in the base media (e.g. RPMI 1640, Biowest, L0498; MCDB131, ThermoFisher, 10372019). Therefore, a significant impact on cell behavior is unlikely. However, residual trace elements in the whey fractions or isolating them from the separated caseins could complement base media lacking trace elements, such as RPMI 1640 (France et al. 2024). Additional lactate and riboflavin from the whey fractions might also improve cell survival, proliferation, or prevent senescence (Zhou et al. 2022; He et al. 2023). However, whether the concentrations measured in this study are effective is unclear. Whey proteins, on the other hand, are a major component of colostrum whey and can serve as a source of amino acids and bioactive proteins (Li et al. 2022; Olvera-Rosales et al. 2023). NanoLC-MS/MS analysis of CW, W, and FCS revealed a variety of proliferation-related proteins, many involved in IGF and TGF-β signaling. Most prominently, IGF-1 was found exclusively in CW, IGF-2 in CW and FCS, and TGF-β2 in W. All of these proteins are known to regulate cell survival, proliferation, and differentiation (Schabort et al. 2009; Aboalola and Han 2017; Li et al. 2017; Yi et al. 2018; Zhang et al. 2018), but their final effects depend heavily on the cellular context and other proteins (Ren et al. 2010; Gardner et al. 2011). IGFs can either promote proliferation and survival via the MAPK pathway or stimulate myogenic differentiation through the PI3K/Akt pathway. Although IGF-1 favors the first pathway and IGF-2 the second, both can act through either pathway depending on factors like timing, concentration and context (Coolican et al. 1997; Yoshiko et al. 2002). TGF-β isoforms have been described to inhibit differentiation and increase proliferation in C2C12 cells. This effect might be mediated by decreased MyoD and increased PCNA stability, influencing their nuclear localization (Schabort et al. 2009). The high dependency on context is further exemplified by the detection of several forms of IGFBPs and myostatin and HTRA1 in CW and W. Although myostatin is associated with inhibiting myoblast growth and differentiation (Thomas et al. 2000; Ge et al. 2020), it can also promote proliferation and transdifferentiation in myoblasts (Rodgers et al. 2014; Uemura et al. 2017). Additionally, its effects can be overruled by IGF-1 (Ren et al. 2010). IGFBPs regulate IGF activity by binding to it, and depending on their isoform and context, they can either enhance or inhibit IGF’s potency, stability, and localization (Ewton and Florini 1995; Silverman et al. 1995; Paye and Forsten-Williams 2006; Witt et al. 2017; Geng et al. 2024). Sometimes, this regulation occurs paradoxically (Yin et al. 2004). The serine-protease HTRA1, on the other hand, can cleave IGFBPs and TGF-β, thus modulating signaling pathways (Eigenbrot et al. 2012; Tiaden and Richards 2013; Li et al. 2020b). Despite the complex context dependency, the combination of IGF-1, IGFBP4, and IGFBP7 makes CW a promising candidate as a media supplement (Witt et al. 2017; Yi et al. 2018; Geng et al. 2024).

Indeed, CW supported cell survival and proliferation in short-term C2C12 culture. W did not show any positive effect on the cell growth. This contrasts with previously published findings of a 1000 kDa milk-derived extract leading to enhanced proliferation in C2C12 cells and bovine myoblasts (Shima et al. 2025). The composition of this whey and W should be similar, however, the composition of the source milk depends on many factors. To name a few, breed, age, nutrition, or lactation stage of the cow, even season or climate, can impact the exact composition of the milk (Alothman et al. 2019).

Notably, single supplementation of lactose, as the main component of CW, did not show any pro-proliferative effect. This underlines that C2C12, as a non-enterocyte mammalian cell type, cannot use lactose as a source of carbon and energy, although exceptions of other cell types metabolizing disaccharides have been described (Leong et al. 2017).

The primary goal of this paper was to prove the applicability of a CW-based serum-free medium for long-term cultures, such as biomass production in cultivated meat. For this purpose, a serum-free medium formulation containing 5 mg/mL CW, 1% ITS, and 1% P/S in RPMI 1640 was developed (CM). Markers of stemness, such as Pax7, are often present in only a subset of cells, expressed ambiguously, and quickly downregulated, making them rather unreliable for assessing the usability of CM. Therefore, a more application-focused readout was chosen by testing CM’s ability to promote long-term proliferation and subsequent myogenic differentiation of the resulting cell population. Indeed, CM supported the culture of C2C12 myoblasts for over 30 days with only slightly slower proliferation compared to the control, which makes prolonged experimental models and scale-up of production processes, for example for cultivated meat, possible. Additionally, long-term culture results in cells that can still undergo myogenic differentiation with the same pattern of myosin expression. Although similar differentiation is observed, the degree of differentiation was lower after cultivation in CM than after 10% FCS. This may be due to insufficient stimulation of differentiation or altered pathways due to CM culture. The most common protocol for differentiation is serum starvation, the deprivation of growth factors. This triggers an intricate sequence of regulation of autocrine-active factors like timely patterned upregulation of IGF-1 and IGF-2 and downregulation of bFGF and TGF-β (Yoshiko et al. 2002; Aboalola and Han 2017). Other relevant targets have been identified as receptors for transferrin and lysophosphatidic acid (Messmer et al. 2022). Activation of insulin/IGF-pathways, transferrin receptors, and inhibition of TGF-β have already been accounted for in the serum-free differentiation medium in this study. It may still be possible to achieve higher degrees of differentiation after CM culture by activation of the LPA receptor or retinoid X receptor and inhibition of the MEK/ERK or NOTCH pathways (Melzener et al. 2024).

Given the necessity of cryopreservation in both laboratory cell culture experiments and industrial cell mass production, evaluating the suitability of CM as a base for a freezing mix was of particular interest. Usually, DMSO is used to prevent harmful crystallization. FCS mitigates the cytotoxic effects of DMSO while also helping to reduce crystallization (Liu et al. 2010). It has already been shown that bovine whey can substitute FCS in freezing mixes and even outperform it in respect to viability (Capiaumont et al. 2000). Consistent with this, a freezing mix with double concentrated CW and 10% DMSO allowed for cryopreservation of cells with higher efficiency as a freezing mix with FCS. This makes CM a versatile medium with an easily applicable freezing method.

To further assess the suitability of CM for tissue engineering applications such as cultivated meat, the medium was also tested in 3D-suspension culture of myospheres. Up to now, C2C12 myospheres have been described not to proliferate, or only negligibly, under static or dynamic culture conditions (Aguanno et al. 2019; Stange et al. 2022; Johnson et al. 2024). The findings of this study regarding compaction, slight proliferation, and viability across all tested spheroid sizes in serum-containing media align with this data. In contrast, CM caused a significantly higher increase in size and DNA content over 14 days, indicating cell proliferation. All initial spheroid sizes grew up to roughly the same limit during this period. Contact inhibition, NOTCH signaling, mimicry of the stem cell niche by ECM, and differentiation have been discussed as reasons for the lack of proliferation of spheroids (Aguanno et al. 2019; Stange et al. 2022; Johnson et al. 2024). CM’s complex composition of bioactive molecules may interfere with these processes. Compared to FCS-containing medium, it might prevent differentiation stronger, which is also suggested by the slightly lower baseline expression of Myog and Myosin measured on day 0 of 2D differentiation in this study. Furthermore, attachment factors present in FCS might be missing in CW and, by that, lead to an alternative microenvironment and signaling within the spheroids, which could explain the slightly lower compaction rate (Da Lee et al. 2022).

While all these hypotheses need to be tested in future research, CM already offers a feasible and more sustainable alternative for FCS in tissue engineering of muscle cells and their scale-up. Colostrum is often regarded as a readily available byproduct of the dairy industry. Accounting for approximately 0.5% of a cow’s total milk output, its production exceeds the consumption by the calf and it cannot be marketed as conventional milk (Kaplan et al. 2021). Repurposing the byproduct colostrum as a supplement for cell culture media is especially beneficial for applications like cultivated meat. Colostrum whey-supplemented media can help achieve the goal of sustainable alternative meat production by avoiding animal slaughter, reducing culture media costs, and promoting waste recycling.

## Conclusion

In this study, CW produced by microfiltration was identified as a potential serum replacement, which is rich in nutrients and growth factors. A CM medium based on CW, RPMI 1640, and ITS was developed for 2D culture of C2C12 myoblasts. CM supported stable growth over 30 days and allowed for subsequent robust differentiation into myotubes. Adaptation of CM into a freezing mix also enabled easy cryopreservation of the cells with higher efficiency than the control. Since scalability and 3D culture are essential for tissue engineering and cultivated meat, myosphere culture in CM was established. Spheroids stayed viable over 14 days. In contrast to their counterparts in FCS-containing media, spheroids in CM grew in size and accumulated DNA, indicating cell proliferation. Overall, this makes CM a promising candidate for sustainable, serum-free culture of myoblasts in biomedical muscle tissue engineering and cultivated meat, repurposing a highly abundant and easily accessible byproduct.

## Supplementary Information

Additional file 1.

## Data Availability

The datasets used and/or analysed during the current study are available from the corresponding author.

## Abbreviations

W: Whey
CW: Colostrum whey
CM: Colostrum whey medium
CMD: Differentiation after colostrum whey medium
FD: Differentiation after FCS-containing medium
FMC: Freezing mix colostrum whey medium
FMF: Freezing mix FCS-containing medium
GDF8: Growth and differentiation factor 8
IGFBP: Insulin-like growth factor binding protein
ITS: Insulin transferrin selenium
IGF: Insulin-like growth factor
TGF: Transforming growth factor
EGF: Epithelial growth factor
FGF: Fibroblast growth factor
FCS: Fetal calf serum
ECM: Extracellular matrix
MYH: Myosin heavy chain
Myog: Myogenin
B2m: Beta 2 microglobulin
