# Supplementary material for: Dairy byproducts as sustainable alternatives to FCS in 2D and 3D skeletal muscle cell cultures
Source: Bioresour Bioprocess. 2025 Sep 22;12(1):101. doi: 10.1186/s40643-025-00938-w (PMC12454707; doi:10.1186/s40643-025-00938-w)
Supplement: Supplementary file 1 — Additional file 1. [file 40643_2025_938_MOESM1_ESM.docx]

# Supplementary Material

**Supplementary Table 1: Quantification of amino acids in FCS, colostrum whey and whey.**

| **in µg/mL** | **His** | **Asn** | **Ser** | **Gln** | **Gly** | **Asp** | **Glu** | **Thr** | **Ala** | **Pro** | **Lys** | **Tyr** | **Met** | **Val** | **IIe** | **Leu** | **Phe** | **Trp** | **Cys** | **Arg** |
| --- | --- | --- | --- | --- | --- | --- | --- | --- | --- | --- | --- | --- | --- | --- | --- | --- | --- | --- | --- | --- |
| **FCS_1** | 6.95 | 3.25 | 15.45 | n.a. | 13.95 | 12.4 | 28.75 | 10.75 | 24.1 | 9.85 | 18.55 | 11.05 | 3.8 | 15.15 | 10.9 | 20 | 12.15 | 4.8 | <LOD | <LOD |
| **FCS_2** | 6.8 | 3.4 | 15.2 | n.a. | 13.55 | 12 | 27.95 | 10.4 | 23.45 | 9.65 | 18.1 | 10.7 | 3.65 | 14.45 | 10.55 | 19.2 | 11.8 | 4.6 | <LOD | <LOD |
| **Colostrum Whey_1** | <LOD | <LOD | <LOD | 31.5 | 7.85 | <LOD | 9.2 | <LOD | 3.8 | 4.3 | 5.8 | 3.5 | <LOD | 6.6 | 3.95 | 5 | <LOD | <LOD | <LOD | <LOD |
| **Colostrum Whey_2** | <LOD | <LOD | <LOD | 29.4 | 7.4 | <LOD | 8.7 | <LOD | 3.65 | 4.15 | 5.6 | 3.55 | <LOD | 6.25 | 3.75 | 4.8 | <LOD | <LOD | <LOD | <LOD |
| **Whey_1** | <LOD | 4.55 | <LOD | 19.95 | 7 | <LOQ | 39.35 | <LOQ | 4.5 | <LOD | 3.3 | 2.45 | <LOD | <LOQ | <LOD | <LOD | <LOD | <LOD | <LOD | <LOD |
| **Whey_2** | <LOD | 4.1 | <LOD | 20.15 | 7.35 | <LOD | 38.85 | <LOQ | 4.6 | <LOD | <LOD | 2.75 | <LOD | <LOQ | <LOD | <LOD | <LOD | <LOD | <LOD | <LOD |

| **in mg/L** | **Ca** | **Cu** | **Fe** | **K** | **Mg** | **Mn** | **Na** | **Zn_Ax** | **Riboflavin** | **Lactate** |
| --- | --- | --- | --- | --- | --- | --- | --- | --- | --- | --- |
| **FCS_1** | 10.82 | <0.1 | <0.5 | 49.16 | 3.37 | <0.1 | 296.88 | 0.22 | 0.08 | 179.90 |
| **FCS_2** | 11.02 | <0.1 | <0.5 | 49.98 | 3.42 | <0.1 | 292.42 | 0.22 | 0.08 | 181.60 |
| **Colostrum Whey_1** | 190.26 | <0.1 | <0.5 | 612.95 | 61.06 | <0.1 | 290.16 | <0.2 | 0.68 | 110.85 |
| **Colostrum Whey_2** | 191.22 | <0.1 | <0.5 | 616.42 | 61.19 | <0.1 | 291.80 | <0.2 | 0.70 | 115.00 |
| **Whey_1** | 227.46 | <0.1 | <0.5 | 1474.91 | 60.99 | <0.1 | 307.93 | <0.2 | 0.15 | 28.74 |
| **Whey_2** | 225.12 | <0.1 | <0.5 | 1476.86 | 60.08 | <0.1 | 306.78 | <0.2 | 0.16 | 28.69 |

**Supplementary Table 2: Composition of RPMI 1640 (L0498, Biowest). DMEM high glucose (L0101, Biowest) and MCDB131 (103372019, Thermo Fisher Scientific).**

| **Component** | **L0498 (mg/L)** |
| --- | --- |
| Glycine | 10 |
| L-Alanyl-L-Glutamine (Glutamine Stable) | 446 |
| L-Arginine Free Base | 200 |
| L-Asparagine Anhydrous | 50 |
| L-Aspartic Acid | 20 |
| L-Cystine Dihydrochloride | 65.2 |
| L-Glutamic Acid | 20 |
| L-Histidine | 15 |
| L-Hydroxy-L-Proline | 20 |
| L-Isoleucine | 50 |
| L-Leucine | 50 |
| L-Lysine Monohydrochloride | 40 |
| L-Methionine | 15 |
| L-Phenylalanine | 15 |
| L-Proline | 20 |
| L-Serine | 30 |
| L-Threonine | 20 |
| L-Tryptophan | 5 |
| L-Tyrosine Disodium Salt Dihydrate | 28.83 |
| L-Valine | 20 |
| Calcium Nitrate Tetrahydrate | 100 |
| Magnesium Sulfate Anhydrous | 48.84 |
| Potassium Chloride | 400 |
| Sodium Bicarbonate | 2000 |
| Sodium Chloride | 6000 |
| Sodium Phosphate Dibasic Anhydrous | 800 |
| Choline Chloride | 3 |
| D-Biotin | 0.2 |
| D-Ca Pantothenate | 0.25 |
| Folic Acid | 1 |
| Myo-Inositol | 35 |
| Nicotinamide (Nicotinic acid amide) | 1 |
| P-Aminobenzoic Acid (PABA) | 1 |
| Pyridoxine Hydrochloride | 1 |
| Riboflavin | 0.2 |
| Thiamine Hydrochloride | 1 |
| Vitamin B12 | 0.005 |
| D-Glucose Anhydrous | 2000 |
| L-Glutathione Reduced | 1 |
| Phenol Red Sodium Salt | 5.3 |

| **Component** | **L0101 (mg/L)** |
| --- | --- |
| Glycine | 30 |
| L-Arginine Monohydrochloride | 84 |
| L-Cystine Dihydrochloride | 62.6 |
| L-Histidine Monohydrochloride Monohydrate | 42 |
| L-Isoleucine | 105 |
| L-Leucine | 105 |
| L-Lysine Monohydrochloride | 146 |
| L-Methionine | 30 |
| L-Phenylalanine | 66 |
| L-Serine | 42 |
| L-Threonine | 95 |
| L-Tryptophan | 16 |
| L-Tyrosine Disodium Salt Dihydrate | 103.79 |
| L-Valine | 94 |
| Calcium Chloride Dihydrate | 265 |
| Ferric Nitrate Nonahydrate | 0.1 |
| Magnesium Sulfate Anhydrous | 97.67 |
| Potassium chloride | 400 |
| Sodium Bicarbonate | 3700 |
| Sodium Chloride | 6400 |
| Sodium Phosphate Monobasic Anhydrous | 109 |
| Choline Chloride | 4 |
| D-Ca Pantothenate | 4 |
| Folic Acid | 4 |
| Myo-Inositol | 7.2 |
| Nicotinamide (Nicotinic acid amide) | 4 |
| Pyridoxal Hydrochloride | 4 |
| Riboflavin | 0.4 |
| Thiamine Hydrochloride | 4 |
| D-Glucose Anhydrous | 4500 |
| Phenol Red Solution Salt | 15.9 |

| **Component** | **103372019 (mg/L)** |
| --- | --- |
| Glycine | 2.3 |
| L-Alanine | 2.7 |
| L-Arginine hydrochloride | 63.2 |
| L-Asparagine-H2O | 15 |
| L-Aspartic acid | 13.3 |
| L-Cysteine 2HCl·H2O | 35 |
| L-Glutamic Acid | 4.4 |
| L-Histidine hydrochloride-H2O | 42 |
| L-Isoleucine | 66 |
| L-Leucine | 131 |
| L-Lysine hydrochloride | 182 |
| L-Methionine | 15 |
| L-Phenylalanine | 33 |
| L-Proline | 11.5 |
| L-Serine | 32 |
| L-Threonine | 12 |
| L-Tryptophan | 4.1 |
| L-Tyrosine | 18.1 |
| L-Valine | 117 |
| Biotin | 0.0073 |
| Choline chloride | 14 |
| D-Calcium pantothenate | 12 |
| Folinic Acid Calcium salt | 0.6 |
| Niacinamide | 6.1 |
| Pyridoxine hydrochloride | 2.1 |
| Riboflavin | 0.0038 |
| Thiamine hydrochloride | 3.4 |
| Vitamin B12 | 0.0136 |

| **Component** | **(mg/mL)** |
| --- | --- |
| i-Inositol | 7.2 |
| Ammonium Molybdate | 0.0037 |
| Ammonium metavanadate | 0.0006 |
| Calcium Chloride | 235 |
| Cupric sulfate | 0.0012 |
| Ferric sulfate | 0.283 |
| Magnesium Sulfate | 2464 |
| Manganese Sulfate | 0.0002 |
| Nickelous Chloride | 7.10E-05 |
| Potassium Chloride | 298 |
| Selenious Acid | 0.0038 |
| Sodium Bicarbonate | 1176 |
| Sodium Chloride | 6430 |
| Sodium Meta Silicate | 2.8 |
| Sodium Phosphate dibasic | 134 |
| Zinc Sulfate | 0.0003 |
| Adenine | 0.135 |
| D-Glucose (Dextrose) | 1000 |
| Lipoic Acid | 0.0021 |
| Phenol Red | 12.4 |

S**upplementary Table 3: Calculated cell numbers after 4 days of culture in DMEM high glucose, RPMI 1640 and MCDB 131 with different supplementations.**

|  | **Whey** | | | | | | **Colostrum Whey** | | | | | |
| --- | --- | --- | --- | --- | --- | --- | --- | --- | --- | --- | --- | --- |
| **DMEM/HG** | 2,612 | 3,982 | 3,617 | 1,053 | 1,098 | 1,330 | 1,692 | 1,923 | 1,874 | 1,150 | 980 | 1,211 |
| **RPMI 1640** | 7,504 | 15,520 | 11,177 | 9,434 | 10,547 | 10,621 | 66,025 | 65,284 | 32,102 | 41,552 | 36,858 | 23,599 |
| **MCDB 131** | 3,962 | 9,409 | 7,913 | 3,756 | 6,128 | 6,274 | 20,711 | 23,809 | 16,486 | 14,113 | 17,817 | 15,541 |
|  | **Whey + Colostrum Whey** | | | | | | **Whey + Colostrum Whey 1/2** | | | | | |
| **DMEM/HG** | 1,617 | 1,837 | 1,760 | 967 | 903 | 926 | 2,073 | 2,430 | 2,057 | 932 | 926 | 886 |
| **RPMI 1640** | 19,210 | 23,684 | 18,008 | 32,096 | 28,373 | 25,898 | 45,010 | 51,643 | 37,159 | 34,379 | 35,054 | 24,196 |
| **MCDB 131** | 21,171 | 22,347 | 17,979 | 13,315 | 15,777 | 15,128 | 24,199 | 29,248 | 17,821 | 9,611 | 11,932 | 12,709 |
|  | **FCS** | | | | | | **w/o** | | | | | |
| **DMEM/HG** | 107,368 | 108,723 | 103,668 | 110,296 | 102,997 | 107,971 | 1,772 | 2,682 | 2,511 | 1,052 | 1,035 | 1,046 |
| **RPMI 1640** | 122,763 | 112,310 | 104,875 | 126,716 | 121,857 | 118,593 | 5,874 | 12,711 | 7,834 | 5,377 | 6,841 | 6,631 |
| **MCDB 131** | 82,239 | 86,723 | 81,656 | 82,621 | 85,389 | 81,177 | 7,651 | 9,651 | 8,077 | 2,718 | 4,229 | 4,009 |

**Supplementary Table 4: Cell numbers seeded (S) and counted at passages in long term proliferation until day 30.**

| **Cell Number x10^5^** | | **S** | **Day 2** | **S** | **Day 5** | **S** | **Day 7** | **S** | **Day 9** | **S** | **Day 12** | **S** | **Day 14** | **S** | **Day 16** | **S** | **Day 19** | **S** | **Day 22** | **S** | **Day 26** | **S** | **Day 28** | **S** | **Day 30** |
| --- | --- | --- | --- | --- | --- | --- | --- | --- | --- | --- | --- | --- | --- | --- | --- | --- | --- | --- | --- | --- | --- | --- | --- | --- | --- |
| **A** | **CW** | 1.70 | 2.63 | 0.50 | 1.45 | 1.45 | 2.69 | 1.50 | 2.45 | 1.00 | 1.29 | 1.29 |  |  |  |  |  |  |  |  |  |  |  |  |  |
|  | **ITS** | 1.70 | 1.32 | 1.20 | 0.22 |  |  |  |  |  |  |  |  |  |  |  |  |  |  |  |  |  |  |  |  |
|  | **CM** | 1.70 | 4.03 | 0.50 | 3.28 | 1.50 | 5.24 | 1.50 | 7.08 | 1.00 | 11.30 | 1.20 | 1.20 | 1.20 | 3.70 | 3.70 | 50.20 | 1.86 | 3.48 | 0.84 | 25.63 | 4.50 | 14.17 | 10.00 | 39.10 |
|  | **FCS** | 1.70 | 7.87 | 0.50 | 8.70 | 1.20 | 4.29 | 1.20 | 8.07 | 0.50 | 11.37 | 1.00 | 3.70 | 2.80 | 20.90 | 0.50 | 5.40 | 0.88 | 19.98 | 0.40 | 11.28 | 2.80 | 9.32 | 7.00 | 42.27 |
|  | **w/o** | 1.70 | 0.27 | 0.27 | 0.25 |  |  |  |  |  |  |  |  |  |  |  |  |  |  |  |  |  |  |  |  |
| **B** | **CW** | 1.70 | 2.39 | 0.50 | 0.31 | 0.31 | 0.10 | 0.10 |  |  |  |  |  |  |  |  |  |  |  |  |  |  |  |  |  |
|  | **ITS** | 1.70 | 0.62 | 0.62 | 0.32 |  |  |  |  |  |  |  |  |  |  |  |  |  |  |  |  |  |  |  |  |
|  | **CM** | 1.70 | 2.76 | 0.50 | 3.89 | 1.50 | 4.19 | 1.50 | 11.18 | 1.00 | 4.06 | 1.20 | 5.38 | 4.50 | 18.90 | 5.00 | 37.07 | 1.86 | 5.33 | 0.84 | 20.30 | 4.50 | 10.97 | 10.00 | 39.70 |
|  | **FCS** | 1.70 | 6.97 | 0.50 | 9.50 | 1.20 | 5.72 | 1.20 | 5.57 | 0.50 | 16.22 | 1.00 | 1.36 | 1.36 | 13.93 | 0.50 | 10.22 | 0.88 | 17.58 | 0.40 | 10.05 | 2.80 | 11.60 | 7.00 | 35.97 |
|  | **w/o** | 1.70 | 0.26 | 0.26 | 0.23 |  |  |  |  |  |  |  |  |  |  |  |  |  |  |  |  |  |  |  |  |
| **C** | **CW** | 1.70 | 2.02 | 0.50 | 0.68 | 0.68 | 0.30 | 0.30 |  |  |  |  |  |  |  |  |  |  |  |  |  |  |  |  |  |
|  | **ITS** | 1.70 | 0.69 | 0.69 | 0.17 |  |  |  |  |  |  |  |  |  |  |  |  |  |  |  |  |  |  |  |  |
|  | **CM** | 1.70 | 3.42 | 0.50 | 1.36 | 1.50 | 7.27 | 1.50 | 3.40 | 1.00 | 14.72 | 1.20 | 1.99 | 1.99 | 8.19 | 5.00 | 51.80 | 1.86 | 4.66 | 0.84 | 19.20 | 4.50 | 13.23 | 10.00 | 37.00 |
|  | **FCS** | 1.70 | 5.10 | 0.50 | 10.22 | 1.20 | 5.03 | 1.20 | 4.73 | 0.50 | 14.03 | 1.00 | 1.25 | 1.25 | 8.44 | 0.50 | 9.02 | 0.88 | 17.43 | 0.40 | 6.57 | 2.80 | 11.83 | 7.00 | 36.50 |
|  | **w/o** | 1.70 | 0.33 | 0.33 | 0.12 |  |  |  |  |  |  |  |  |  |  |  |  |  |  |  |  |  |  |  |  |


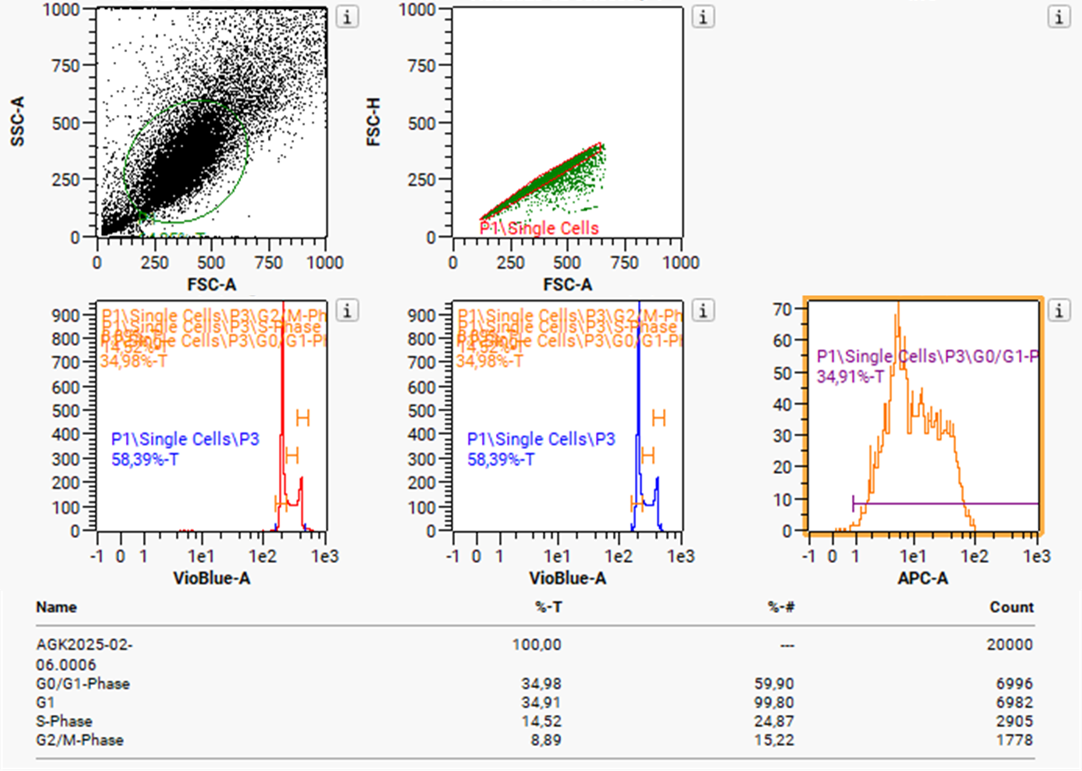


**Supplementary Figure 1: Exemplary gating and analysis of the cell cycle analysis by FACS.**


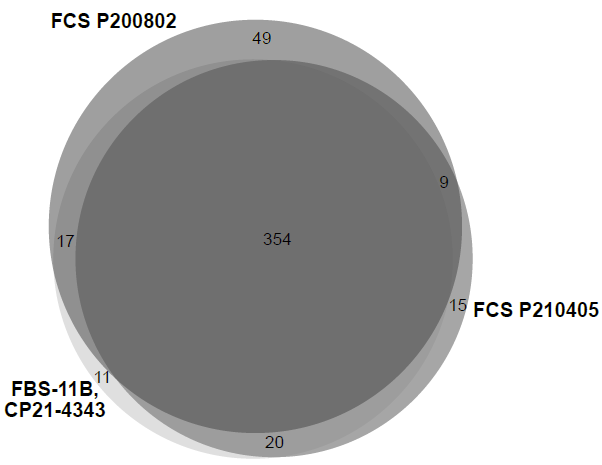


**Supplementary Figure 2: Total protein overlaps of different FCS batches.** FCS batches used in mass spectrometry measurements showed minor differences pointing to negligible batch variabilities.


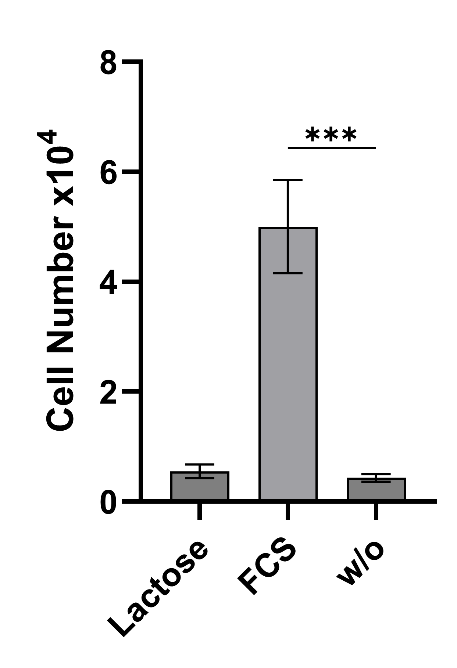


**Supplementary Figure 3: Short-term culture of C2C12 cells in RPMI 1640 supplemented with lactose.** To simulate lactose levels under colostrum whey supplementation 4 mg/mL lactose were used. No significant differences between lactose supplementation and negative control (w/o) were found. *** p ≤ 0.001. n=3.


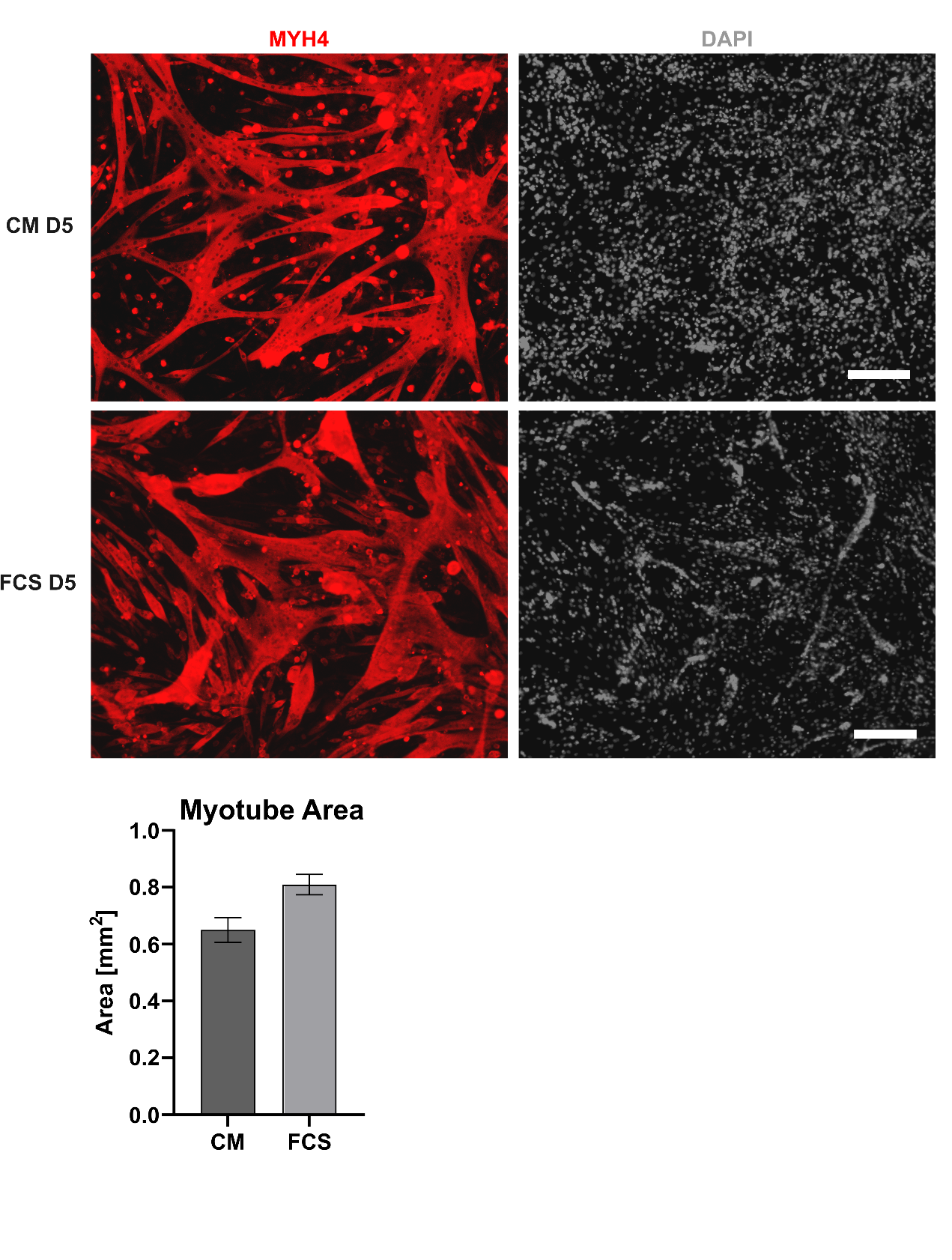


**Supplementary Figure 4: Myogenic differentiation after 30 days of culture in CM or FCS-containing medium.** Immunofluorescence stainings against MYH4 at 5 days after differentiation start. Myotube area was measured as the area of MYH4 positive area per picture. n=3.


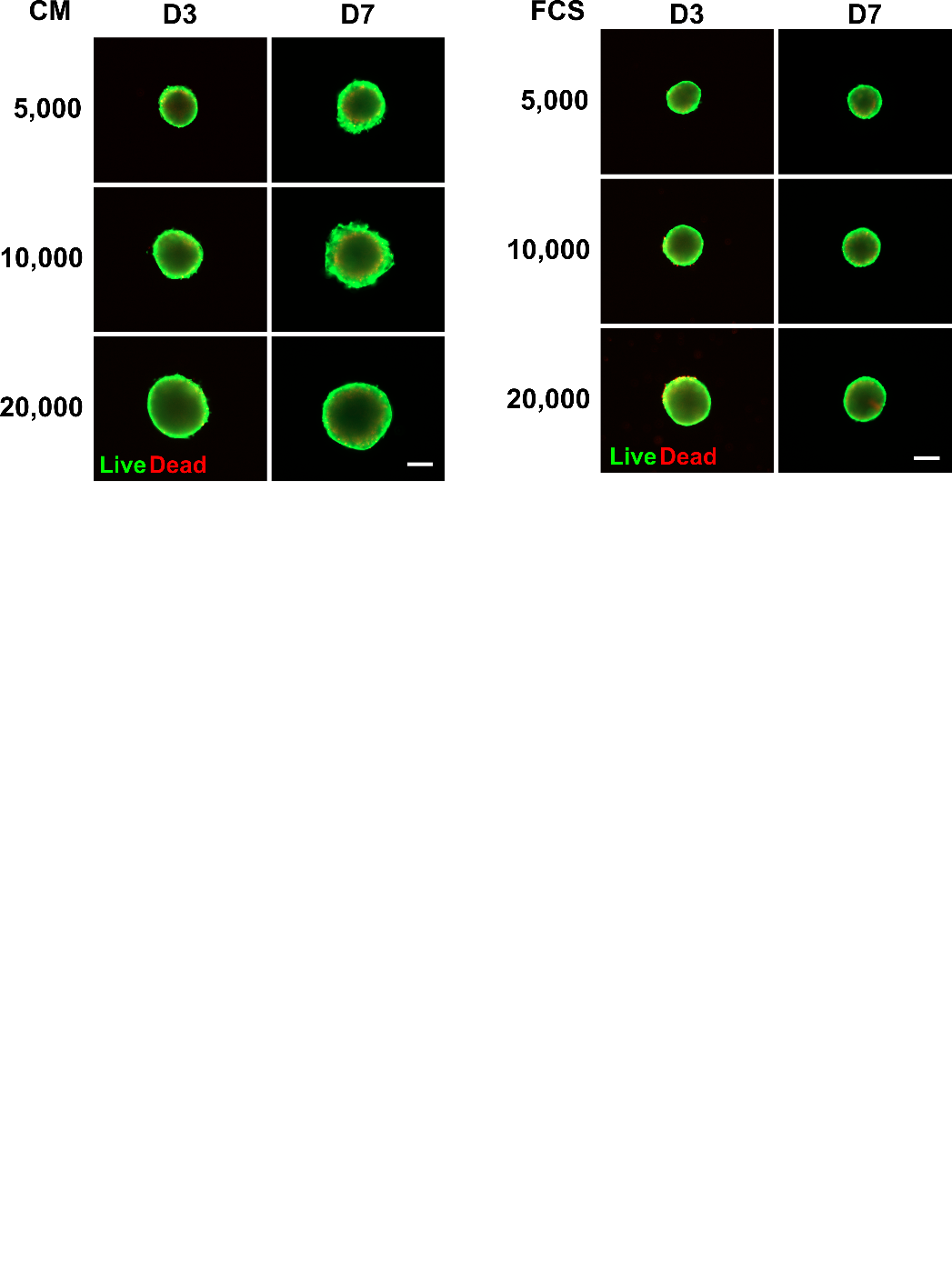


**Supplementary Figure 5: Viability staining of spheroids on day 3 and 7 in all conditions.**

**Supplementary Table 5: Measured area of spheroids in phase contrast images.**

| **5000 cells** |  |  |  |  |  |  |  |  |  |  |  | in µm^2 |
| --- | --- | --- | --- | --- | --- | --- | --- | --- | --- | --- | --- | --- |
|  | **D1** | | | **D3** | | | **D7** | | | **D14** | | |
| **CM** | 77,678.05 | 80,715.30 | 83,248.13 | 57,822.85 | 64,435.60 | 66,069.94 | 103,997.37 | 98,196.95 | 83,605.47 | 318,400.90 | 384,986.22 | 410,936.22 |
| **FCS 10%** | 71,271.54 | 62,585.73 | 66,072.58 | 52,014.89 | 47,173.34 | 51,871.81 | 49,079.43 | 50,780.19 | 48,125.00 | 57,692.59 | 106,387.19 | 69,218.14 |
|  |  |  |  |  |  |  |  |  |  |  |  |  |
|  |  |  |  |  |  |  |  |  |  |  |  |  |
| **10000 cells** |  |  |  |  |  |  |  |  |  |  |  |  |
|  | **D1** | | | **D3** | | | **D7** | | | **D14** | | |
| **CM** | 131,697.37 | 144,427.08 | 141,651.25 | 91,008.80 | 101,093.98 | 96,691.27 | 119,377.42 | 122,470.50 | 127,537.40 | 330,579.50 | 238,338.02 | 277,726.59 |
| **FCS 10%** | 108,735.80 | 98,940.72 | 103,480.96 | 74,356.02 | 65,031.79 | 68,788.50 | 67,226.18 | 62,147.16 | 60,643.14 | 98,521.40 | 126,041.48 | 82,750.62 |
|  |  |  |  |  |  |  |  |  |  |  |  |  |
|  |  |  |  |  |  |  |  |  |  |  |  |  |
| **20000 cells** |  |  |  |  |  |  |  |  |  |  |  |  |
|  | **D1** | | | **D3** | | | **D7** | | | **D14** | | |
| **CM** | 217,507.83 | 246,412.81 | 255,735.94 | 138,403.81 | 148,119.81 | 150,439.13 | 175,270.15 | 158,645.85 | 162,233.80 | 439,840.24 | 367,902.42 | 478,457.00 |
| **FCS 10%** | 183,885.25 | 173,567.66 | 168,278.53 | 106,952.56 | 100,204.92 | 94,580.61 | 88,984.21 | 106,803.81 | 74,914.68 | 139,280.54 | 164,912.88 | 125,777.22 |

**Supplementary Table 6: Relative fluorescence units of DAPI-DNA-quantification of spheroids.**

| **5000 cells** |  |  |  |  |  |  |  |  |  | in relative flourescence units | | |
| --- | --- | --- | --- | --- | --- | --- | --- | --- | --- | --- | --- | --- |
|  | **D1** | | | **D3** | | | **D7** | | | **D14** | | |
| **CM** | 190,735.30 | 192,599.80 | 240,805.30 | 203,574.13 | 212,921.55 | 221,182.80 | 299,845.55 | 401,529.80 | 369,885.47 | 778,951.80 | 921,987.80 | 739,115.55 |
| **FCS 10%** | 249,804.55 | 209,027.30 | 213,494.55 | 234,052.05 | 211,664.80 | 198,268.05 | 190,014.55 | 205,909.30 | 184,952.55 | 254,742.30 | 278,239.47 | 461,615.30 |
|  |  |  |  |  |  |  |  |  |  |  |  |  |
|  |  |  |  |  |  |  |  |  |  |  |  |  |
| **10000 cells** |  |  |  |  |  |  |  |  |  |  |  |  |
|  | **D1** | | | **D3** | | | **D7** | | | **D14** | | |
| **CM** | 284,233.80 | 226,147.80 | 270,502.55 | 279,225.55 | 326,807.30 | 293,018.05 | 440,360.55 | 348,562.05 | 332,830.30 | 807,481.55 | 858,977.05 | 741,700.05 |
| **FCS 10%** | 256,419.80 | 249,127.80 | 273,688.30 | 320,245.80 | 236,173.55 | 242,435.80 | 349,699.80 | 374,459.55 | 275,342.05 | 397,504.13 | 374,094.80 | 459,352.80 |
|  |  |  |  |  |  |  |  |  |  |  |  |  |
|  |  |  |  |  |  |  |  |  |  |  |  |  |
| **20000 cells** |  |  |  |  |  |  |  |  |  |  |  |  |
|  | **D1** | | | **D3** | | | **D7** | | | **D14** | | |
| **CM** | 289,193.55 | 292,813.80 | 305,705.30 | 332,289.30 | 303,048.05 | 409,343.30 | 491,340.55 | 430,706.05 | 432,499.55 | 602,210.05 | 756,051.55 | 1,042,390.55 |
| **FCS 10%** | 268,669.80 | 243,444.80 | 390,295.55 | 268,486.30 | 339,408.55 | 360,104.30 | 275,703.55 | 413,020.05 | 546,331.47 | 452,222.55 | 479,337.30 | 419,109.05 |
|  |  |  |  |  |  |  |  |  |  |  |  |  |
